# Estimating Dementia Risk Using Multifactorial Prediction Models

**DOI:** 10.1001/jamanetworkopen.2023.18132

**Published:** 2023-06-13

**Authors:** Mika Kivimäki, Gill Livingston, Archana Singh-Manoux, Nina Mars, Joni V. Lindbohm, Jaana Pentti, Solja T. Nyberg, Matti Pirinen, Emma L. Anderson, Aroon D. Hingorani, Pyry N. Sipilä

**Affiliations:** 1 Department of Mental Health of Older People, UCL Brain Sciences, University College London, London, United Kingdom; 2 Clinicum, Faculty of Medicine, University of Helsinki, Helsinki, Finland; 3 Université Paris Cité, Inserm U1153, Epidemiology of Ageing and Neurodegenerative Diseases, Paris, France; 4 Institute for Molecular Medicine, University of Helsinki, Helsinki, Finland; 5 Broad Institute of MIT and Harvard, Cambridge, Massachusetts; 6 Department of Mathematics and Statistics, University of Helsinki, Helsinki, Finland; 7 MRC Integrative Epidemiology Unit and Population Health Sciences, University of Bristol Medical School, Bristol, United Kingdom; 8 Institute of Cardiovascular Sciences, University College London, London, United Kingdom

## Abstract

**Importance:**

The clinical value of current multifactorial algorithms for individualized assessment of dementia risk remains unclear.

**Objective:**

To evaluate the clinical value associated with 4 widely used dementia risk scores in estimating 10-year dementia risk.

**Design, Setting, and Participants:**

This prospective population-based UK Biobank cohort study assessed 4 dementia risk scores at baseline (2006-2010) and ascertained incident dementia during the following 10 years. Replication with a 20-year follow-up was based on the British Whitehall II study. For both analyses, participants who had no dementia at baseline, had complete data on at least 1 dementia risk score, and were linked to electronic health records from hospitalizations or mortality were included. Data analysis was conducted from July 5, 2022, to April 20, 2023.

**Exposures:**

Four existing dementia risk scores: the Cardiovascular Risk Factors, Aging and Dementia (CAIDE)-Clinical score, the CAIDE–*APOE*-supplemented score, the Brief Dementia Screening Indicator (BDSI), and the Australian National University Alzheimer Disease Risk Index (ANU-ADRI).

**Main Outcomes and Measures:**

Dementia was ascertained from linked electronic health records. To evaluate how well each score predicted the 10-year risk of dementia, concordance (C) statistics, detection rate, false-positive rate, and the ratio of true to false positives were calculated for each risk score and for a model including age alone.

**Results:**

Of 465 929 UK Biobank participants without dementia at baseline (mean [SD] age, 56.5 [8.1] years; range, 38-73 years; 252 778 [54.3%] female participants), 3421 were diagnosed with dementia at follow-up (7.5 per 10 000 person-years). If the threshold for a positive test result was calibrated to achieve a 5% false-positive rate, all 4 risk scores detected 9% to 16% of incident dementia and therefore missed 84% to 91% (failure rate). The corresponding failure rate was 84% for a model that included age only. For a positive test result calibrated to detect at least half of future incident dementia, the ratio of true to false positives ranged between 1 to 66 (for CAIDE–*APOE*-supplemented) and 1 to 116 (for ANU-ADRI). For age alone, the ratio was 1 to 43. The C statistic was 0.66 (95% CI, 0.65-0.67) for the CAIDE clinical version, 0.73 (95% CI, 0.72-0.73) for the CAIDE–*APOE*-supplemented, 0.68 (95% CI, 0.67-0.69) for BDSI, 0.59 (95% CI, 0.58-0.60) for ANU-ADRI, and 0.79 (95% CI, 0.79-0.80) for age alone. Similar C statistics were seen for 20-year dementia risk in the Whitehall II study cohort, which included 4865 participants (mean [SD] age, 54.9 [5.9] years; 1342 [27.6%] female participants). In a subgroup analysis of same-aged participants aged 65 (±1) years, discriminatory capacity of risk scores was low (C statistics between 0.52 and 0.60).

**Conclusions and Relevance:**

In these cohort studies, individualized assessments of dementia risk using existing risk prediction scores had high error rates. These findings suggest that the scores were of limited value in targeting people for dementia prevention. Further research is needed to develop more accurate algorithms for estimation of dementia risk.

## Key Points

**Question:**

What is the clinical value associated with current multifactorial algorithms in estimating 10-year dementia risk?

**Findings:**

In this cohort study including 465 929 participants from the UK Biobank, 4 widely-used risk scores (Cardiovascular Risk Factors, Ageing and Dementia [CAIDE-Clinical], CAIDE–*APOE*-supplemented, Brief Dementia Screening Indicator [BDSI], and Australian National University Alzheimer Disease Risk Index [ANU-ADRI]) missed 84% to 91% of participants with incident dementia when the threshold for a positive test result was calibrated to achieve a 5% false-positive rate, and to detect at least half of participants with incident dementia, the ratio of true to false positives exceeded 1 to 66. These numbers were better, with 84% of incident dementia missed or a true to false positives ratio of 1 to 43, when estimating dementia risk based on age alone.

**Meaning:**

These findings suggest that current risk scores have limited clinical utility for estimation of 10-year dementia risk.

## Teaser

This cohort study evaluates the clinical value associated with 4 widely used dementia risk scores in estimating 10-year dementia risk among participants in the UK Biobank.

## Introduction

Alzheimer disease and other dementias are a leading cause of mortality and are associated with considerable health and social care costs.^1^ Globally, almost 60 million people live with dementia. With populations aging, the number of people with dementia is projected to more than double by 2050.^2^ There is a pressing need for effective dementia prevention.^3^

To allow targeted prevention, a number of multifactorial risk prediction models have been developed.^4,5,6^ They aim to distinguish people at high risk of dementia, ie, the group who may benefit most from preventive actions, from those at low risk. Some prediction models are widely used in research and clinical trials. The Cardiovascular Risk Factors, Aging, and Dementia (CAIDE) score, designed to predict late-life dementia based on midlife factors. CAIDE (which is also available as a mobile application),^4^ has the following components: age group, sex, education, systolic blood pressure, high body mass index (BMI), total cholesterol, and physical activity; additional information on apolipoprotein E (*APOE*) genotype can be included (CAIDE–*APOE*-supplemented).^7^ The Brief Dementia Screening Indicator (BDSI) is a 7-item weighted instrument that includes age group, education, BMI, depressive symptoms, stroke, diabetes, and requiring assistance with money or medication.^5^ The Australian National University Alzheimer Disease Risk Index (ANU-ADRI) is a self-report instrument that includes 11 risk and 4 protective items (risk: age group, sex, low education, high BMI, total cholesterol, diabetes, traumatic brain injury, depressive symptoms, smoking, low social networks, and occupational pesticide exposure; protective: cognitively stimulating activities, alcohol consumption, physical activity, and fish intake).^6^ Unlike CAIDE scores, the BDSI and ANU-ADRI risk scores have been developed using risk factor data on older adults. In epidemiological studies, concordance (C) statistics for the 4 widely used multifactorial prediction models varied between 0.48 and 0.78, depending on the model and cohort.^5,6,7,8,9,10^ Although higher C statistics have been reported for some new algorithms, these lack validation in independent study populations and are not currently used in health care settings.^11^

For a prediction model to aid clinical decision-making, 2 important but often unreported measures of performance are the detection rate, which denotes the proportion of individuals with a positive test result among people who developed the disease at follow-up, and false-positive rate, ie, the proportion of people with a positive test result among those who did not develop the disease at follow up.^12^ Defining an appropriate threshold for a positive test result (ie, the score above which a patient is allocated to the high-risk group to recommend intervention) requires consideration of trade-offs between detection and false-positive rates. Poor detection rate implies that a large number of people who will develop dementia are misinformed about their high risk. A high false-positive rate, in turn, means that many individuals who will not develop dementia are informed that they are at high risk, potentially resulting in unnecessary distress.

We used these metrics to evaluate the clinical value of CAIDE, CAIDE–*APOE*-supplemented, BDSI, and ANU-ADRI scores in estimating 10-year dementia risk. As research on CAIDE originally used midlife risk factors for prediction of old-age dementia, we also evaluated the value of this risk score in estimating 20-year risk of dementia. For comparison, we estimated the accuracy of risk assessment using information on a person’s age alone, the simplest manner to target dementia prevention in the population.

## Methods

This cohort study was approved by the North-West Multi-Centre Research Ethics Committee, the University College London Hospital Committee on the Ethics of Human Research, and the London–Harrow Research Ethics Committee. Participants provided written informed consent at each contact. This study followed the Strengthening the Reporting of Observational Studies in Epidemiology (STROBE) reporting guideline for cohort studies.

### Study Design and Oversight

This observational study was based on 2 prospective cohort studies, the UK Biobank study and the Whitehall II study. For estimation of 10-year dementia risk, we used data from the UK Biobank study, a nationwide study of half a million participants aged between 38 and 73 years and living in the UK.^13^ Participants volunteered in 21 assessment centers across England, Wales, and Scotland using standardized procedures. Baseline clinical examinations, including measures for the assessment of dementia risk, were conducted between March 13, 2006, and October 1, 2010. Participant follow-up via linked electronic health records of the UK National Health Service (NHS) started at baseline and ended on September 30, 2021, in England; July 31, 2021, in Scotland; and February 28, 2018, in Wales. The NHS provides most of the health care in the UK, including inpatient and outpatient care, and record linkage is undertaken using a unique NHS identifier held by all UK residents.

**Figure 1.**
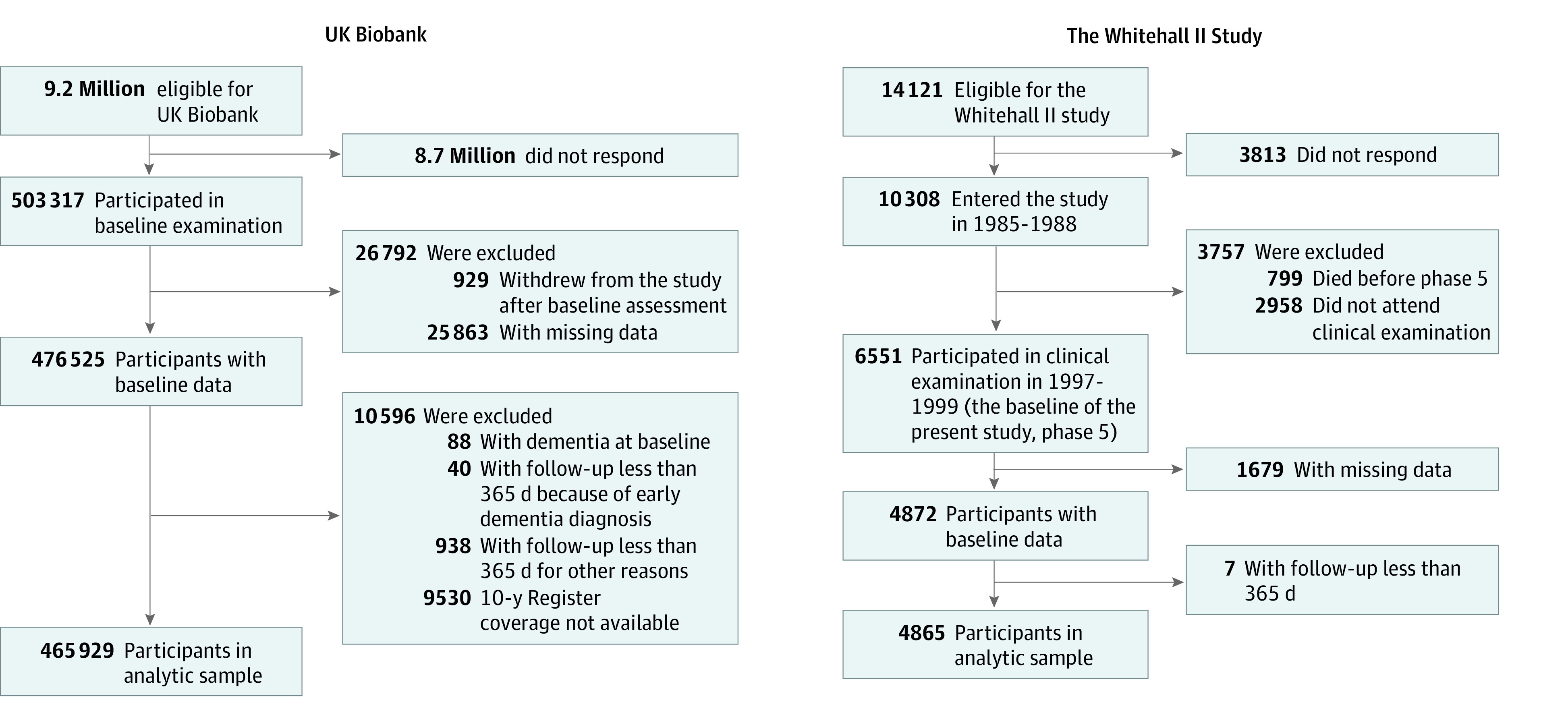
Selection of Study Participants in the UK Biobank and Whitehall II Studies

Selection of participants for this study is shown in Figure 1. We included participants who did not have dementia at baseline, had complete data on at least 1 of the dementia risk scores, and were linked to electronic health records from hospitalizations or mortality. We excluded participants from Wales whose baseline assessment was less than 10 years before the end of the availability of the hospital records. This study was conducted using the UK Biobank Resource under Application Numbers 60565 and 22627 (data accessed December 9, 2022).

In supplementary analysis for CAIDE, data on midlife risk factors and old-age dementia were obtained from the Whitehall II study of 10 308 UK civil servants, established in 1985 to 1988.^14^ Baseline assessments of dementia risk factors were conducted between April 24, 1997, and January 8, 1999, when participants were aged 45 to 69 years. Participants were linked to electronic health records of the NHS, and data from linked records were updated on an annual basis for 20 years, until October 2, 2019. As in the main analysis, we included participants who did not have dementia at baseline, had complete data on at least 1 of the dementia risk scores, and were linked to electronic health records from hospitalizations or mortality (Figure 1).

### Baseline Data and Dementia Follow-up

Baseline assessments included all components of the CAIDE, BDSI, and ANU-ADRI scores, measured according to standard operating protocols, as detailed in the eMethods in Supplement 1. To characterize the study populations, we measured age, sex and ethnicity. Ethnicity was self-defined as Asian or Asian British, Black or Black British, multiple, White (all with subcategories), or other in UK Biobank and as Black, South Asian, White or other in the Whitehall II study. Dementia was ascertained using primary and secondary diagnoses from linked hospital admission data and from underlying and contributory causes of death being dementia (eMethods in Supplement 1). A diagnosis of all-cause dementia consisted of the following *International Statistical Classification of Diseases and Related Health Problems, Tenth Revision *(*ICD-10*) codes: F00 to F03, F05.1, G30, G31.0, G31.1, and G31.8.^15^ In addition, we considered the following subtypes of dementia: Alzheimer disease (*ICD-10* codes F00 and G30), vascular dementia (*ICD-10* code F01), frontotemporal dementia (*ICD-10* codes F02.0 and G31.0), and Parkinson disease dementia (*ICD-10* code F02.3).^15^

### Statistical Analysis

To exclude prevalent dementia from the analysis and participants with potentially compromised capacity to accurately respond to self-administered risk questionnaires due to preclinical or undiagnosed dementia at the time of risk assessment, we excluded participants with a record of hospitalization due to dementia at baseline (88 participants [0.02%] of all baseline participants in UK Biobank; no participants in the Whitehall II study) or within 12 months after baseline (40 participants [0.01%] in the UK Biobank; no participants in the Whitehall II study).

We determined 4 dementia risk scores (CAIDE clinical version, CAIDE–*APOE*-supplemented version, BDSI, and ANU-ADRI) for each participant using standard formulas and analyzed each risk score separately, excluding participants with missing data on any of the items in the risk score under investigation (eTable 1 in Supplement 1). To mimic clinical practice, we did not impute missing data. Comparisons of dementia risk scores and baseline characteristics between participants who did and did not develop dementia at follow-up were performed using *t* tests or χ^2^ tests, as appropriate.

To evaluate how well each score predicted the 10-year risk of dementia, we computed the C statistic (also known as Harrell C index) for the continuous scores, which in the context of 10-year risk is identical to the area under the receiver operating characteristic curve (AUC) statistic (the probability that an affected individual drawn at random has a higher risk score than an unaffected individual drawn at random). We used Cox proportional hazards models to calculate hazard ratios and related 95% CIs separately for the 4 risk scores and age alone with incident dementia. The scores were divided into quintiles, with the bottom quintile being the reference group.

To evaluate the scores’ clinical value and ability to detect participants who would develop dementia, we dichotomized the risk scores into positive test result vs negative test result using alternative cutoffs. For each risk score, we calculated detection rate, false-positive rate, the number of participants with incident dementia but with a negative test result per 10 participants with incident dementia, and the ratio of true to false positives (eMethods in Supplement 1). For comparison, we calculated these indices for a model including age alone. To further examine the role of age in dementia prognosis, we calculated detection rate, number missed per 10 participants with incident dementia, false-positive rate, and ratio of true to false positives for a subgroup of participants at age 64 to 66 years.

As the CAIDE scores are designed for long-term prediction of dementia in middle-aged people, we tested 20-year risk prediction in the Whitehall II study. Risk scores were assessed at midlife and incident dementia was assessed over the follow-up.

We used Stata MP statistical software version 17 (StataCorp) for data analyses. Syntax for the analyses is provided in the eMethods in Supplement 1. *P* values were 2-sided, and statistical significance was set at *P* = .05. Data analysis was conducted from July 5, 2022, to April 20, 2023.

## Results

A total of 465 929 UK Biobank participants (mean [SD] age, 56.5 [8.1] years; range, 38-73 years; 252 778 [54.3%] female participants) did not have dementia at baseline, had complete data on at least 1 of the dementia risk scores, were linked to electronic health records from hospitalizations or mortality, and had at least 1 year of follow-up (Figure 1). Table 1 shows the baseline characteristics of the participants divided into whether they did or did not develop dementia by follow-up.

**Table 1.** Characteristics of Participants at Baseline

Characteristic	All	Dementia	No dementia	*P* value
**UK Biobank, 10-y follow-up**				
Sample size, No.	465 929	3421	462 508	NA
Age, mean (SD), y	56.5 (8.1)	64.2 (4.9)	56.4 (8.1)	<.001
Sex, No. (%)				
Male	213 151 (45.7)	1839 (53.8)	211 312 (45.7)	<.001
Female	252 778 (54.3)	1582 (46.2)	251 196 (54.3)	
Ethnicity, No. (%)				
Asian or Asian British, Black or Black British, or other or multiple ethnicities	23 313 (5.0)	136 (4.0)	23 177 (5.0)	.02
White	441 140 (94.7)	3273 (95.7)	437 867 (94.7)	
Unknown	1476 (0.3)	12 (0.4)	1464 (0.3)	
Dementia risk score, mean (SD)				
CAIDE (clinical version)	6.2 (2.7)	7.7 (2.0)	6.2 (2.7)	<.001
CAIDE (*APOE* supplemented)	7.3 (3.2)	9.8 (2.5)	7.3 (3.2)	<.001
BDSI	4.6 (5.4)	8.4 (6.5)	4.6 (5.4)	<.001
ANU-ADRI	−4.4 (6.1)	−2.3 (6.9)	−4.4 (6.1)	<.001
**Whitehall II study, 20-y follow-up**				
Sample size, No.	4865	202	4663	NA
Age, mean (SD), y	54.9 (5.9)	61.2 (4.3)	55.2 (6.0)	<.001
Sex, No. (%)				
Male	3524 (72.4)	136 (67.3)	3387 (72.6)	.10
Female	1342 (27.6)	66 (32.7)	1276 (27.4)	
Ethnicity, No. (%)				
Black, South Asian, other, or did not respond	353 (7.2)	26 (12.9)	327 (7.0)	.002
White	4512 (92.7)	176 (87.1)	4336 (93.0)	
Dementia risk score, mean (SD)				
CAIDE (clinical version)	6.1 (2.0)	7.1 (1.8)	6.1 (1.9)	<.001
CAIDE (*APOE* supplemented)	6.9 (2.3)	8.9 (2.2)	6.8 (2.3)	<.001

Abbreviations: ANU-ADRI, Australian National University Alzheimer Disease Risk Index; BDSI, Brief Dementia Screening Indicator; CAIDE, Cardiovascular Risk Factors, Aging, and Dementia Score; NA, not applicable.

**Figure 2.**
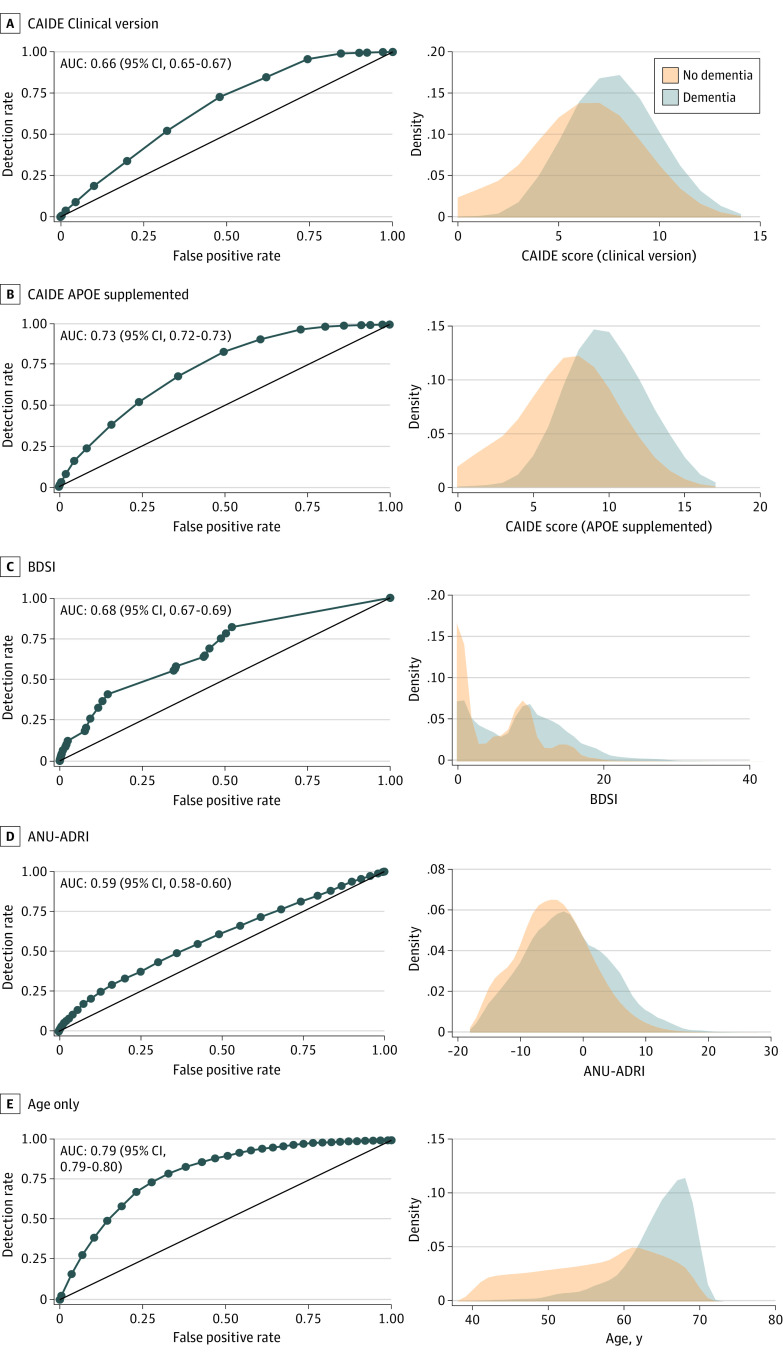
Area Under Curve (AUC) and Distributions for Risk Scores and Age by Incident Dementia ANU-ADRI indicates Australian National University Alzheimer Disease Risk Index; BDSI, Brief Dementia Screening Indicator; CAIDE, Cardiovascular Risk Factors, Aging, and Dementia Score.

During 10 years of follow-up, 3421 UK Biobank participants (0.7%) were diagnosed with dementia (7.5 per 10 000 person-years). The distributions of dementia risk scores were highly overlapping in participants with and without incident dementia, although mean scores differed (Figure 2). The associations between risk score quintiles and dementia incidence followed a dose-response pattern, but the gradient was steeper using only information from participant’s age (eTable 2 in Supplement 1).

As shown in Table 2, C statistics for all-cause dementia varied between 0.59 (95% CI, 0.58-0.60) for ANU-ADRI and 0.73 (95% CI, 0.72-0.73) for CAIDE–*APOE*-supplemented, but it was higher (C = 0.79; 95% CI, 0.79-0.80) for the model including only age. C statistics for dementia subtypes were also highest using only information on participant’s age, ranging from 0.68 (95% CI, 0.63-0.72) to 0.82 (95% CI, 0.80-0.83), followed by CAIDE–*APOE*-supplemented, ranging from 0.64 (95% CI, 0.58-0.69) to 0.76 (95% CI, 0.74-0.77). For all dementia risk scores and age alone, the highest C statistic was observed for vascular dementia and the lowest for frontotemporal dementia.

**Table 2.** C Statistic for Dementia Risk Scores by Dementia Subtype

Dementia subtype by risk score	C (95% CI)
**CAIDE Clinical version**	
All-cause dementia	0.66 (0.65-0.67)
Alzheimer disease	0.66 (0.65-0.67)
Vascular dementia	0.70 (0.68-0.72)
Frontotemporal dementia	0.61 (0.57-0.66)
Parkinson disease dementia	0.63 (0.60-0.67)
**CAIDE APOE supplemented**	
All-cause dementia	0.73 (0.72-0.73)
Alzheimer disease	0.74 (0.73-0.76)
Vascular dementia	0.76 (0.74-0.77)
Frontotemporal dementia	0.64 (0.58-0.69)
Parkinson disease dementia	0.69 (0.65-0.73)
**BDSI**	
All-cause dementia	0.68 (0.67-0.69)
Alzheimer disease	0.68 (0.66-0.69)
Vascular dementia	0.73 (0.71-0.75)
Frontotemporal dementia	0.59 (0.54-0.65)
Parkinson disease dementia	0.66 (0.61-0.70)
**ANU-ADRI**	
All-cause dementia	0.59 (0.58-0.60)
Alzheimer disease	0.57 (0.55-0.59)
Vascular dementia	0.64 (0.61-0.67)
Frontotemporal dementia	0.54 (0.47-0.61)
Parkinson disease dementia	0.55 (0.49-0.60)
**Age only**	
All-cause dementia	0.79 (0.79-0.80)
Alzheimer disease	0.81 (0.80-0.82)
Vascular dementia	0.82 (0.80-0.83)
Frontotemporal dementia	0.68 (0.63-0.72)
Parkinson disease dementia	0.81 (0.78-0.84)

Abbreviations: ANU-ADRI, Australian National University Alzheimer Disease Risk Index; BDSI, Brief Dementia Screening Indicator; CAIDE, Cardiovascular Risk Factors, Aging, and Dementia Score.

Table 3 shows detection rate, number missed per 10 participants with incident dementia, false-positive rate, and the ratio of true to false positives for dichotomized dementia risk scores using alternative cutoffs for positive test results. Incident dementia numbers are presented in eTable 3 in Supplement 1. Defining the test-positive threshold to detect more than 50% of incident dementia led to a false-positive rate of 32% and a true to false positives ratio of 1 to 88 for CAIDE clinical, a false-positive rate of 24% and a true to false positives ratio of 1 to 66 for CAIDE–*APOE*-supplemented, a false-positive rate of 34% and a true to false positives ratio of 1 to 87 for BDSI, and a false-positive rate of 43% and a true to false positives ratio of 1 to 116 for ANU-ADRI. A test-positive threshold that kept the false-positive rate at or below 5%, in turn, missed 8.4 to 9.1 per 10 participants with incident dementia, depending on the risk score. Risk assessment based on age alone had a lower error rate. To detect more than 50% of incident dementia, the threshold for a test-positive result was at age 65 years, yielding a false-positive rate of 19% and a true to false positives ratio of 1 to 43. For a false-positive rate at or below 5%, the threshold for a test-positive result was at age 69 years, and 8.4 in 10 participants with incident dementia were missed.

**Table 3.** Capacity of Dementia Risk Scores and Age Only to Estimate 10-Year Dementia Risk

Risk score and alternative cutoffs	Detection rate	Missed per 10 participants with incident dementia	False-positive rate	True to false positives ratio
**CAIDE clinical version (observed, 0 to 14)**				
0-5 vs ≥6	0.85	1.5	0.62	1 to 104
0-6 vs ≥7	0.73	2.7	0.48	1 to 94
0-7 vs ≥8	0.52	4.8	0.32	1 to 88
0-8 vs ≥9^a^	0.34	6.6	0.20	1 to 85
0-9 vs ≥10^a^	0.19	8.1	0.10	1 to 77
0-10 vs ≥11	0.09	9.1	0.05	1 to 72
0-11 vs ≥12	0.04	9.6	0.02	1 to 59
**CAIDE–*APOE*-supplemented (observed, 0 to 17)**				
0-5 vs ≥6	0.97	0.3	0.73	1 to 107
0-6 vs ≥7	0.91	0.9	0.61	1 to 96
0-7 vs ≥8	0.83	1.7	0.50	1 to 85
0-8 vs ≥9	0.68	3.2	0.36	1 to 75
0-9 vs ≥10^a^	0.52	4.8	0.24	1 to 66
0-10 vs ≥11^a^	0.38	6.2	0.16	1 to 59
0-11 vs ≥12	0.24	7.6	0.08	1 to 51
0-12 vs ≥13	0.16	8.4	0.05	1 to 41
0-13 vs ≥14	0.08	9.2	0.02	1 to 38
**BDSI (observed, 0 to 42)**				
0-4 vs ≥5	0.65	3.5	0.44	1 to 95
0-6 vs ≥7	0.58	4.2	0.35	1 to 85
0-8 vs ≥9	0.55	4.5	0.34	1 to 87
0-9 vs ≥10	0.41	5.9	0.15	1 to 50
0-14 vs ≥15	0.18	8.2	0.08	1 to 58
0-15 vs ≥16	0.12	8.8	0.02	1 to 28
0-21 vs ≥22^a^	0.03	9.7	0.004	1 to 17
**ANU-ADRI (observed, −18 to 28)**				
≤−11 vs ≥−10	0.88	1.2	0.84	1 to 142
≤−6 vs ≥−5	0.66	3.4	0.56	1 to 126
≤−4 vs ≥−3	0.55	4.5	0.43	1 to 116
≤−3 vs ≥−2	0.49	5.1	0.36	1 to 110
≤0 vs ≥1	0.33	6.7	0.20	1 to 92
≤4 vs ≥5	0.17	8.3	0.08	1 to 66
≤5 vs ≥6	0.13	8.7	0.06	1 to 64
≤6 vs ≥7	0.11	8.9	0.04	1 to 61
≤9 vs ≥10	0.05	9.5	0.02	1 to 45
**Age alone (observed, 38-73), y**				
≤49 vs ≥50	0.98	0.2	0.76	1 to 105
≤54 vs ≥55	0.95	0.5	0.61	1 to 87
≤59 vs ≥60	0.86	1.4	0.43	1 to 67
≤63 vs ≥64	0.68	3.2	0.23	1 to 46
≤64 vs ≥65	0.59	4.1	0.19	1 to 43
≤65 vs ≥66	0.49	5.1	0.14	1 to 39
≤66 vs ≥67	0.39	6.1	0.10	1 to 36
≤67 vs ≥68	0.28	7.2	0.07	1 to 33
≤68 vs ≥69	0.16	8.4	0.04	1 to 30
≤69 vs ≥70	0.02	9.8	0.005	1 to 26

Abbreviations: ANU-ADRI, Australian National University Alzheimer Disease Risk Index; BDSI, Brief Dementia Screening Indicator; CAIDE, Cardiovascular Risk Factors, Aging, and Dementia Score.

^a^ Cutoffs recommended in literature.

In subgroups of middle-aged (aged ≤64 years at baseline) and older (aged ≥65 years at baseline) participants, the performance of the risk scores was similar or worse than in the total sample (eTable 4 and eTable 5 in Supplement 1). The findings did not change after including incident dementia from the first year of follow-up (eTable 6 in Supplement 1).

To minimize the effect of age on predictive model performance, we conducted a sensitivity analysis in a subgroup of participants aged 65 (± 1) years. The predictive capacity of all risk scores was attenuated in this same-aged population. C statistics ranged from 0.52 (95% CI, 0.50-0.54) to 0.60 (95% CI, 0.58-0.62) (eTable 7 in Supplement 1).

Additional analyses were based on the Whitehall II study (Table 1). Estimates for 20-year dementia prediction in this younger cohort of 4865 participants were not materially different from those in the main analysis, except that the true to false positives ratio improved owing to a greater proportion of participants with incident dementia in this cohort (eTable 8 in Supplement 1). For the CAIDE clinical score, the C statistic was 0.65 (95% CI, 0.61-0.68), the true to false positives ratio at 50% detection rate was 1 to 16, and the number of missed participants with incident dementia at 5% false-positive rate was 9.1 per 10 participants with incident dementia. The corresponding metrics were a C statistic of 0.74 (95% CI, 0.70-0.78), a true to false positives ratio of 1 to 10, and 9.0 missed participants per 10 participants with incident dementia for the CAIDE–*APOE*-supplemented score. Risk assessment was more accurate using age alone (C = 0.79; 95% CI, 0.77-0.82; true to false positives ratio at 50% detection rate, 1 to 9; missed cases at 5% false-positive rate, 9.1 per 10 participants with incident dementia).

## Discussion

In this cohort study, all dementia prediction models investigated were characterized by a high false-positive rate for higher detection rates and by low detection rates when the false-positive rate was kept low. To detect half of future dementia cases using the CAIDE, BDSI, and ANU-ADRI scores, each correct prediction of dementia was accompanied by 66 to 116 false-positive predictions. If the test-positive threshold was calibrated to provide a low false-positive rate (≤5%), then these scores missed 84% to 91% of incident dementia. In a group of same-aged individuals aged 65 (±1) years, the C statistic was between 0.52 and 0.60, indicating that the models predicted dementia only marginally better than chance. These data suggest that population stratification and individualized assessment of dementia risk using existing prediction algorithms have high error rates.

The C statistics for the CAIDE clinical version (0.66), CAIDE–*APOE*-supplemented version (0.73), BDSI (0.68), and ANU-ADRI (0.59) in UK Biobank are comparable with those reported in other studies,^6,7,8,9,10^ although higher C statistics have been found in the cohorts used to develop these algorithms. The AUC was 0.77 for CAIDE clinical version in the derivation cohort of 1400 middle-aged adults followed up for 20 years,^7^ but C statistics were lower (0.49-0.71) in subsequent validation analyses using independent populations.^6,8^ C statistics for the BDSI ranged from 0.68 to 0.78 in the cohorts used for the development of this measure,^5^ and C statistics for ANU-ADRI ranged from 0.67 to 0.77 in studies with short follow-ups (<10 years).^5,6,9^ In 4 cohorts of patients with stroke, the C statistic was 0.61 for the BDSI and 0.66 for ANU-ADRI, both performing better than CAIDE clinical version (AUC, 0.53).^10^ None of these studies reported key indices for clinical decision-making, such as detection rate at specific false-positive rates and ratios of true to false positives.

The clinical value of a prediction model is partly dependent on the available preventive intervention. In the absence of disease-modifying drugs, lifestyle modification, social engagement, and control for cardiometabolic risk factors (eg, hypertension and diabetes) are considered the best available options to prevent or delay the onset of dementia.^16,17,18,19^ Although these interventions are safe for most people, a false-positive test result in dementia risk assessment may not be without harm, as it elicits psychological distress for affected individuals due to implied possibility of developing an incurable disease. The ratio of detected incident dementia to false-positive results was poor, at 1 to 77 or 1 to 85 for the most-widely used CAIDE clinical version using recommended thresholds.^20^ For comparison, this ratio is 1 to 10 or better in prediction of 10-year cardiovascular disease risk using the US Pooled Cohort Equation^21^ and 1 to 1.5 in 4-year prediction of cardiovascular outcomes using 27 plasma proteins.^22,23^

Minimizing the false-positive rate in dementia prediction by raising the threshold for a test-positive result is not without problems. Risk algorithms calibrated at a 5% false-positive rate missed 8 to 9 of 10 participants who developed incident dementia. With this calibration, most people who will develop dementia would not be informed about their high risk or the need to take preventive measures. Risk assessment with low detection to minimize the false-positive rate is acceptable for new interventions with uncertain safety profiles or interventions with limited availability. Neither of these conditions apply to current dementia prevention.

Aging increases the susceptibility to a wide range of diseases, including dementia.^24^ We found that using age alone to assess dementia risk outperformed the 4 risk scores. This favors reliance on population-wide strategies and campaigns targeted at all older people above a certain age, although age-based risk stratification does not inform early interventions for middle-aged individuals.

### Limitations

This study has some limitations. Generalizability of this study is unknown. UK Biobank and Whitehall II participants were healthier and with more favorable levels of risk factors than the UK general population.^25,26^ Nonetheless, in the context of associations of risk factors with disease, the findings from these 2 cohorts are in close agreement with those from studies that are more representative of the general population.^25,26^ This suggests that our results on the clinical utility of the 4 dementia risk scores might apply to the UK general population. While 85.5% of individuals who were invited participated in the examination of dementia risk factors in the Whitehall II study, participation was only 5.5% in the UK Biobank cohort. Low participation might contribute to overestimation or underestimation of true predictive capacity, although substantial bias is more likely to be introduced by a large number of dropouts during follow-up, which was avoided in both studies due to use of linked outcome data.^25^

The wording of questions in the UK Biobank survey did not exactly match those in the BDSI and the ANU-ADRI, and this could have affected our findings. We used linkage to electronic health records from high-coverage national registries to define dementia. It is possible that not all incident dementia was captured in these records, but previous research suggests high sensitivity and specificity and that use of linked data may have little effect on risk factor associations.^27^ The CAIDE scores were designed for 20-year rather than 10-year prediction of dementia.^7^ While data for long-term follow-up were not available in UK Biobank, a supplementary analysis using a 20-year follow-up of the Whitehall II study participants suggested a similar or worse risk stratification for CAIDE compared with age alone.

## Conclusions

Based on the findings of this cohort study using data from the UK Biobank and Whitehall II studies, we would not advocate implementation of individualized dementia risk assessment using these dementia prediction models. Further research is needed to develop better risk prediction algorithms for dementia. Ideally, risk markers used in algorithms would be surrogate markers responsive to change in risk (unlike age, sex, and *APOE* genotype), as such markers could inform clinical decisions on individualized preventive strategies, a goal increasingly adopted in modern biomarker-based risk prediction tools for chronic conditions.^21,22,28,29^

## Notes

Supplement 1.
eMethods.
**eTable 1.** Proportion of Missing Data for Each Risk Score Item **eTable 2.** Associations Between Dementia Risk Scores and Dementia Incidence **eTable 3.** Capacity of Dementia Risk Scores and Age Only to Estimate 10-Year Dementia Risk **eTable 4.** Capacity of Dementia Risk Scores and Age Only to Estimate 10-Year Dementia Risk Among Participants Aged ≤64 Years **eTable 5.** Capacity of Dementia Risk Scores and Age Only to Estimate 10-Year Dementia Risk Among Participants Aged 65 Years or Older **eTable 6.** Capacity of Dementia Risk Scores and Age Only to Estimate 10-Year Dementia Risk in All Participants Including Those with Follow-up Less Than 365 Days **eTable 7.** Performance of Dementia Risk Scores and Age Only to Estimate 10-Year Dementia Risk at Age 65 (±1) Years **eTable 8.** Capacity of Dementia Risk Scores and Age Only to Estimate 20-Year Dementia Risk in the Whitehall II Study

Supplement 2.
Data Sharing Statement
