# Supplementary material for: Estimating Dementia Risk Using Multifactorial Prediction Models
Source: JAMA Netw Open. 2023 Jun 13;6(6):e2318132. doi: 10.1001/jamanetworkopen.2023.18132 (PMC10265307; doi:10.1001/jamanetworkopen.2023.18132)
Supplement: Supplement 1. — eMethods. eTable 1. Proportion of Missing Data for Each Risk Score Item eTable 2. Associations Between Dementia Risk Scores and Dementia Incidence eTable 3. Capacity of Dementia Risk Scores and Age Only to Estimate 10-Year Dementia Risk eTable 4. Capacity of Dementia Risk Scores and Age Only to Estimate 10-Year Dementia Risk Among Participants Aged ≤64 Years eTable 5. Capacity of Dementia Risk Scores and Age Only to Estimate 10-Year Dementia Risk Among Participants Aged 65 Years or Older eTable 6. Capacity of Dementia Risk Scores and Age Only to Estimate 10-Year Dementia Risk in All Participants Including Those with Follow-up Less Than 365 Days eTable 7. Performance of Dementia Risk Scores and Age Only to Estimate 10-Year Dementia Risk at Age 65 (±1) Years eTable 8. Capacity of Dementia Risk Scores and Age Only to Estimate 20-Year Dementia Risk in the Whitehall II Study [file jamanetwopen-e2318132-s001.pdf]

Supplemental Online Content

Kivimäki M, Livingston G, Singh-Manoux A, et al. Estimating dementia risk using multifactorial prediction models. *JAMA Netw Open*. 2023;6(6):e2318132. doi:10.1001/jamanetworkopen.2023.18132

eMethods.
eTable 1. Proportion of Missing Data for Each Risk Score Item
eTable 2. Associations Between Dementia Risk Scores and Dementia Incidence
eTable 3. Capacity of Dementia Risk Scores and Age Only to Estimate 10-Year Dementia Risk
eTable 4. Capacity of Dementia Risk Scores and Age Only to Estimate 10-Year Dementia Risk Among Participants Aged ≤64 Years
eTable 5. Capacity of Dementia Risk Scores and Age Only to Estimate 10-Year Dementia Risk Among Participants Aged 65 Years or Older
eTable 6. Capacity of Dementia Risk Scores and Age Only to Estimate 10-Year Dementia Risk in All Participants Including Those with Follow-up Less Than 365 Days
eTable 7. Performance of Dementia Risk Scores and Age Only to Estimate 10-Year Dementia Risk at Age 65 (±1) Years
eTable 8. Capacity of Dementia Risk Scores and Age Only to Estimate 20-Year Dementia Risk in the Whitehall II Study

This supplemental material has been provided by the authors to give readers additional information about their work.

© 2023 Kivimäki M et al. *JAMA Network Open.*

Measurement of Dementia Risk Scores

Baseline assessment included all factors in the CAIDE, BDSI and ANU-ADRI scores,[1-4] measured according to standard operating protocols: Age group at recruitment; sex; educational qualifications (converted to years of education); systolic blood pressure (mmHg, automated reading supplemented by manual reading by a trained nurse in UK Biobank and measured with an OMRON HEM 907 machine in the Whitehall II study after 5 minutes of rest); weight (kg); standing height (cm), serum total cholesterol (using an enzymatic assay on a Beckman Coulter AU5800 in UK Biobank and the CHOD–PAP method in the Whitehall II study), physical activity (times per week for CAIDE; weekly metabolic equivalent minutes, converted to International Physical Activity Questionnaire activity groups for ANU-ADRI), diabetes (self-reported diabetes or insulin medication or serum glycated haemoglobin [HbA1c, measured using High Performance Liquid Chromatography] ≥48 mmol/mol), mention of stroke in hospital records (ICD-10 codes I60; I61, I63, I64), need of help from others to manage money or medications (self-reported attendance allowance used as a proxy), self-report of recent symptoms of depression (depressed mood, unenthusiasm/disinterest, or tiredness/lethargy), mention of traumatic brain injury in hospital records (ICD-10 code S06), self-report of smoking, alcohol intake, social engagement (relationship status, participation in pub or social club, frequency of friends/family visits, the existence of a confidant relationship and number of living in household apart from spouse), cognitive activity (adult education class, attending a religious group or other group activity), fish intake and occupational pesticide exposure. In UK Biobank, *APOE* genotype was obtained by Affymetrix using a bespoke BiLEVE Axiom array or the UK Biobank Axiom array and was quality controlled by the Wellcome Trust Centre for Human Genetics (WTCHG), University of Oxford, before release into UK Biobank. In the Whitehall II study, *APOE* genotype was determined using a standard PCR assay (heteroduplex generator) of DNA extracted from whole blood using the salting-out method.[5] *APOE* genotype was directly genotyped based on two SNPs (rs7412 and rs429358). *APOE4*+ dominant model of E2/E4, E3/E4 and E4/E4 was used to define the presence of *APOE4*. None of the risk scores included questions on use of antihypertensive or lipid-lowering medications and the only antidiabetic pharmacotherapy requested was insulin treatment.

Linear combinations to determine dementia risk scores are as follows:

The CAIDE score (Clinical version) = age (<47 = 0 points, 47-53 = 3 points, >53 = 4 points) + years of education (≥10 = 0 points; 7–9 = 2 points, 0–6 = 3 points) + sex (female = 0 points, male = 1 point) + systolic blood pressure (≤140mmHg = 0 point, >140mmHg = 2 points) + BMI (≤30 = 0 points, >30 = 2 points) + total cholesterol (≤6.5mmol/l = 0 points, >6.5mmol/l = 2 points) + physical activity (active = 0 points, inactive = 1 point). Range 0–15

The CAIDE score (APOE supplemented) = Age (<47 = 0 points, 47-53 = 3 points, >53 = 5 points) + Years of education (≥10 = 0 points; 7–9 = 3 points, 0–6 = 4 points) + Sex (female = 0 points, male = 1 point) + Systolic blood pressure (≤140mmHg = 0 point, >140mmHg = 2 points) + BMI (≤30 = 0 points, >30 = 2 points) + Total cholesterol (≤6.5mmol/l = 0 points, >6.5mmol/l = 1 points) + Physical activity (active = 0 points, inactive = 1 point) + APOE4 genotype (non epsilon-4 = 0 points, epsilon4 = 2 points). Range 0–18.

The BDSI = Age (1 point per year above age 65 until age 79) + Years of education (≥12 = 0 points, <12 = 9 points) + BMI (≥18.5 = 0 points, <18.5 = 8 points) + Diabetes (no = 0 points, yes = 3 points) + Stroke (no = 0 points, yes = 6 points) + Help from others to manage money or medications (no = 0 point, yes = 10) + Symptoms of depression (no = 0, yes = 6). Range 0–56.

© 2023 Kivimäki M et al. *JAMA Network Open.*

The ANU-ADRI = Age (for males: <65 = 0 points, 65-69 = 1 point, 70-74 = 12 points, 75-79 = 18 points, 80-84 = 26 points, 85-90 = 33 points, >90 = 38 points; for females: <65 = 0 points, 65-69 = 5 points, 70-74 = 14 points, 75-79 = 21 points, 80-84 = 29 points, 85-90 = 35 points, >90 = 41 points) + Education (>11 years = 0 points, 8-11 years = 3 points, <8 years = 6 points) + BMI (if age <60, <25 = 0 points, 25- <30 = 2 points, ≥30 = 5 points) + diabetes (no = 0 points, yes = 3 points) + symptoms of depression (no = 0 points, yes = 2 points) + total cholesterol (if age <60, <6.2mmol/l = 0 points, ≥6.2mmol/l = 3 points) + traumatic brain injury (no = 0 points, yes = 4 points) + smoking (never = 0 points, past = 1 point, current = 4 points) + alcohol intake (non-drinker = 0 points, drinker = -3 points) + social engagement (high = 0 points, medium to high = 1 point, low to medium = 4 points, low = 6 points) + physical activity (IPAQ-low = 0 points, IPAQ-moderate = -2 points, IPAQ-high = -3 points) + Cognitive activity (low = 0 points, medium = -7 points [sic], high = -6 points [sic]) + fish intake (<0.25 portions/week = 0 points, 0.25 - <2 portions/week = -3 points, 2-4 portions/week = -4 points, >4 portions/week = -5 points) + occupational pesticide exposure (rarely or never = 0 points, more often = 2 points). Range -18 – 114.

Ascertainment of Dementia

Dementia was ascertained using primary and secondary diagnoses from linked hospital admission data from Hospital Episode Statistics–Admitted Patient Care (HES APC England), SMR01 (Scottish Morbidity Records–General/Acute Inpatient and Day Case Admissions [Scotland]) and PEDW (Patient Episode Database for Wales) and from underlying/contributory causes of death being dementia. A diagnosis of all-cause dementia consisted of the following International Classification of Diseases, 10th Revision (ICD-10) codes: F00–F03, F05.1, G30, G31.0, G31.1, and G31.8.[6] In addition, we considered the following subtypes of dementia: Alzheimer's disease (F00, G30), vascular dementia (F01), frontotemporal dementia (F02.0, G31.0), and Parkinson's disease dementia (F02.3).[6] In the Whitehall II study, only 3-digit codes for dementia diagnosis were available: F00-F03, G30, G31.

Assessment of clinical value

To evaluate the score's clinical value and ability to detect those who would develop dementia, we dichotomized the risk scores into 'test positive' versus 'test negative' using alternative cut-offs. For each risk score, we calculated detection rate (the proportion of incident dementia cases who were test positive, also known as sensitivity), false positive rate (the proportion of non-dementia cases who nevertheless were test positive, which equals to 1 - specificity), the number of dementia cases missed per 10 cases, and the ratio of true to false positives (also referred to as the odds of being affected given a positive result [OAPR]). The formulas were as follows:

$$\text{Detection rate} = a/(a+c)$$
$$\text{Number missed per 10 cases} = c/(a + c) * 10$$
$$\text{False positive rate} = b/(b+d)$$
$$\text{Ratio true-to-false positives} = a/b, \text{ expressed as 1-to-}(b/a),$$

where a, b, c and d represent different combinations of risk scores and incident dementia as defined below:

	DEMENTIA DURING THE FOLLOW-UP	
RISK SCORE	Yes	No
Test positive	a	b
Test negative	c	d

© 2023 Kivimäki M et al. *JAMA Network Open.*

References

1. Sindi S, Calov E, Fokkens J, et al. The CAIDE Dementia Risk Score App: The development of an evidence-based mobile application to predict the risk of dementia. Alzheimers Dement (Amst) 2015;1(3):328-33.

2. Barnes DE, Beiser AS, Lee A, et al. Development and validation of a brief dementia screening indicator for primary care. Alzheimer's & dementia : the journal of the Alzheimer's Association 2014;10(6):656-65.

3. Anstey KJ, Cherbuin N, Herath PM, et al. A self-report risk index to predict occurrence of dementia in three independent cohorts of older adults: the ANU-ADRI. PLoS One 2014;9(1):e86141.

4. Kivipelto M, Ngandu T, Laatikainen T, et al. Risk score for the prediction of dementia risk in 20 years among middle aged people: a longitudinal, population-based study. The Lancet Neurology 2006;5(9):735-41.

5. Miller SA, Dykes DD, Polesky HF. A simple salting out procedure for extracting DNA from human nucleated cells. Nucleic Acids Res 1988;16(3):1215.

6. Sipilä PN, Heikkilä N, Lindbohm JV, et al. Hospital-treated infectious diseases and the risk of dementia: a large, multicohort, observational study with a replication cohort. Lancet Infectious Diseases 2021;21(11):1557-67.

© 2023 Kivimäki M et al. *JAMA Network Open.*

eTable 1. Proportion of Missing Data for Each Risk Score Item

Variable	N (%)
Age at entry, years	
Available	>502383 (100.0%)
Missing	<5 (0.0%)
Sex	
Available	502388 (100.0%)
Ethnicity	
Available	499609 (99.4%)
Missing	2779 (0.6%)
Education, years	
Available	492256 (98.0%)
Missing	10132 (2.0%)
Systolic blood pressure	
Available	500554 (99.6%)
Missing	1834 (0.4%)
Body mass index	
Available	499281 (99.4%)
Missing	3107 (0.6%)
Total cholesterol	
Available	469480 (93.4%)
Missing	32908 (6.6%)
Physical activity, times per week	
Available	474813 (94.5%)
Missing	27575 (5.5%)
Physical activity, IPAQ group	
Available	402283 (80.1%)
Missing	100105 (19.9%)
APOE4 carrier	
Available	413037 (82.2%)
Missing	89351 (17.8%)
Diabetes mellitus	
Available	468501 (93.3%)
Missing	33887 (6.7%)
History of stroke	
Available	502388 (100.0%)
Needs help	
Available	496856 (98.9%)
Missing	5532 (1.1%)
Depressive symptoms	
Available	466469 (92.9%)
Missing	35919 (7.1%)
History of traumatic brain injury	
Available	502388 (100.0%)
Smoking status	
Available	499438 (99.4%)
Missing	2950 (0.6%)
Alcohol drinking	
Available	500886 (99.7%)
Missing	1502 (0.3%)
Social engagement	
Available	477212 (95.0%)
Missing	25176 (5.0%)
Cognitive activity	
Available	499553 (99.4%)
Missing	2835 (0.6%)
Fish intake	
Available	496638 (98.9%)
Missing	5750 (1.1%)
Occupational pesticide exposure	
Available	502388 (100.0%)

Abbreviations: IPAQ, International Physical Activity Questionnaire.

© 2023 Kivimäki M et al. *JAMA Network Open.*

eTable 2. Associations Between Dementia Risk Scores and Dementia Incidence

Risk score	Quintile	Range	N	N dementia (%)	HR (95% CI)
CAIDE Score	1st	0 to 4	109105	130 (0.1)	1 (reference)
(Clinical version)	2nd	5 to 6	113225	683 (0.6)	5.14 (4.26 to 6.20)
	3rd	7	68017	611 (0.9)	7.72 (6.39 to 9.33)
	4th	8 to 9	94527	994 (1.1)	9.12 (7.60 to 10.95)
	5th	10 to 14	43537	560 (1.3)	11.38 (9.40 to 13.77)
CAIDE Score	1st	0 to 5	95944	76 (0.08)	1 (reference)
(APOE supplemented)	2nd	6 to 7	83368	345 (0.4)	5.29 (4.13 to 6.79)
	3rd	8	49577	377 (0.8)	9.80 (7.66 to 12.54)
	4th	9 to 10	72687	747 (1.0)	13.37 (10.56 to 16.93)
	5th	11 to 17	57540	957 (1.7)	22.03 (17.44 to 27.83)
BDSI	1st and 2nd	0	199139	530 (0.3)	1 (reference)
	3rd	1 to 6	70921	712 (1.0)	3.85 (3.44 to 4.31)
	4th	7 to 9	86076	506 (0.6)	2.23 (1.97 to 2.52)
	5th	10 to 42	61432	1207 (2.0)	7.76 (7.00 to 8.59)
ANU-ADRI	1st	-18 to -10	66912	329 (0.5)	1 (reference)
	2nd	-9 to -6	77640	410 (0.5)	1.07 (0.93 to 1.24)
	3rd	-5 to -3	63580	373 (0.6)	1.20 (1.03 to 1.39)
	4th	-2 to 1	65237	432 (0.7)	1.36 (1.18 to 1.57)
	5th	2 to 28	53685	635 (1.2)	2.48 (2.17 to 2.83)
Age only	1st	38 to 48	96303	50 (0.05)	1 (reference)
	2nd	49 to 55	99868	169 (0.2)	3.29 (2.40 to 4.51)
	3rd	56 to 60	91340	353 (0.4)	7.59 (5.64 to 10.20)
	4th	61 to 64	90169	845 (0.9)	18.57 (13.96 to 24.70)
	5th	65 to 73	88249	2004 (2.3)	46.33 (34.99 to 61.34)

Abbreviations: ANU-ADRI, the Australian National University Alzheimer's Disease Risk Index; BDSI, the Brief Dementia Screening Indicator; CAIDE Score, the Cardiovascular Risk Factors, Aging, and Dementia Score; CI, confidence interval; HR, hazard ratio.

© 2023 Kivimäki M et al. *JAMA Network Open.*

eTable 3. Capacity of Dementia Risk Scores and Age Only to Estimate 10-Year Dementia Risk

Risk score	Observed range	Alternative cut offs	Dementia		No dementia		Detection rate	Number missed per 10 cases	False positive rate	Ratio true-to-false positives
			Test pos	Test neg	Test pos	Test neg				
CAIDE Score	0 to 14									
(Clinical version)		0-5 vs ≥6	2522	456	263 517	161 916	0.85	1.5	0.62	1 to 104
		0-6 vs ≥7	2165	813	203 916	221 517	0.73	2.7	0.48	1 to 94
		0-7 vs ≥8	1554	1424	136 510	288 923	0.52	4.8	0.32	1 to 88
		0-8 vs ≥9*	1009	1969	85 349	340 084	0.34	6.6	0.20	1 to 85
		0-9 vs ≥10*	560	2418	42 977	382 456	0.19	8.1	0.10	1 to 77
		0-10 vs ≥11	268	2710	19 404	406 029	0.09	9.1	0.05	1 to 72
		0-11 vs ≥12	114	2864	6 714	418 719	0.04	9.6	0.02	1 to 59
CAIDE Score	0 to 17									
(APOE supplemented)		0-5 vs ≥6	2426	76	260 746	95 868	0.97	0.3	0.73	1 to 107
		0-6 vs ≥7	2274	228	217 353	139 261	0.91	0.9	0.61	1 to 96
		0-7 vs ≥8	2081	421	177 723	178 891	0.83	1.7	0.50	1 to 85
		0-8 vs ≥9	1704	798	128 523	228 091	0.68	3.2	0.36	1 to 75
		0-9 vs ≥10*	1307	1195	86 326	270 288	0.52	4.8	0.24	1 to 66
		0-10 vs ≥11*	957	1545	56 583	300 031	0.38	6.2	0.16	1 to 59
		0-11 vs ≥12	593	1909	29 979	326 635	0.24	7.6	0.08	1 to 51
		0-12 vs ≥13	399	2103	16 487	340 127	0.16	8.4	0.05	1 to 41
		0-13 vs ≥14	197	2305	7 422	349 192	0.08	9.2	0.02	1 to 38
BDSI	0 to 42									
		0-4 vs ≥5	1910	1045	181 945	232 668	0.65	3.5	0.44	1 to 95
		0-6 vs ≥7	1713	1242	145 795	268 818	0.58	4.2	0.35	1 to 85
		0-8 vs ≥9	1636	1319	142 900	271 713	0.55	4.5	0.34	1 to 87
		0-9 vs ≥10	1207	1748	60 225	354 388	0.41	5.9	0.15	1 to 50
		0-14 vs ≥15	541	2414	31 463	383 150	0.18	8.2	0.08	1 to 58
		0-15 vs ≥16	362	2593	10 026	404 587	0.12	8.8	0.02	1 to 28
		0-21 vs ≥22*	96	2859	1 615	412 998	0.03	9.7	0.004	1 to 17
ANU-ADRI	-18 to 28									
		≤-11 vs ≥-10	1917	262	271 328	53 547	0.88	1.2	0.84	1 to 142
		≤-6 vs ≥-5	1440	739	181 062	143 813	0.66	3.4	0.56	1 to 126
		≤-4 vs ≥-3	1192	987	138 608	186 267	0.55	4.5	0.43	1 to 116
		≤-3 vs ≥-2	1067	1112	117 855	207 020	0.49	5.1	0.36	1 to 110
		≤0 vs ≥1	721	1458	66 024	258 851	0.33	6.7	0.20	1 to 92
		≤4 vs ≥5	375	1804	24 679	300 196	0.17	8.3	0.08	1 to 66
		≤5 vs ≥6	293	1886	18 797	306 078	0.13	8.7	0.06	1 to 64
		≤6 vs ≥7	229	1950	13 888	310 987	0.11	8.9	0.04	1 to 61
		≤9 vs ≥10	117	2062	5 291	319 584	0.05	9.5	0.02	1 to 45
Age only	38 to 73									
		≤49 vs ≥50	3363	58	353 101	109 407	0.98	0.2	0.76	1 to 105
		≤54 vs ≥55	3239	182	282 204	180 304	0.95	0.5	0.61	1 to 87
		≤59 vs ≥60	2952	469	198 199	264 309	0.86	1.4	0.43	1 to 67
		≤63 vs ≥64	2312	1109	106 715	355 793	0.68	3.2	0.23	1 to 46
		≤64 vs ≥65	2004	1417	86 245	376 263	0.59	4.1	0.19	1 to 43
		≤65 vs ≥66	1693	1728	66 095	396 413	0.49	5.1	0.14	1 to 39
		≤66 vs ≥67	1330	2091	47 825	414 683	0.39	6.1	0.10	1 to 36
		≤67 vs ≥68	958	2463	31 381	431 127	0.28	7.2	0.07	1 to 33
		≤68 vs ≥69	549	2872	16 401	446 107	0.16	8.4	0.04	1 to 30
		≤69 vs ≥70	85	3336	2 173	460 335	0.02	9.8	0.005	1 to 26

Abbreviations: ANU-ADRI, the Australian National University Alzheimer's Disease Risk Index; BDSI, the Brief Dementia Screening Indicator; CAIDE Score, the Cardiovascular Risk Factors, Aging, and Dementia Score.
*Cut offs recommended in literature.

© 2023 Kivimäki M et al. *JAMA Network Open.*

eTable 4. Capacity of Dementia Risk Scores and Age Only to Estimate 10-Year Dementia Risk Among Participants Aged ≤64 Years

Risk score	Observed range	C-index (95% CI)	Alternative cut offs	Dementia		No dementia		Detection rate	Number missed per 10 cases	False positive rate	Ratio true-to-false positives
				Test pos	Test neg	Test pos	Test neg				
CAIDE Score	0 to 14	0.66 (0.65 to 0.68)									
(Clinical version)			0-5 vs ≥6	994	254	196 624	151 370	0.80	2.0	0.57	1 to 198
			0-6 vs ≥7	821	427	147 268	200 726	0.66	3.4	0.42	1 to 179
			0-7 vs ≥8	581	667	96 211	251 783	0.47	5.3	0.28	1 to 166
			0-8 vs ≥9*	369	879	58 622	289 372	0.30	7.0	0.17	1 to 159
			0-9 vs ≥10*	204	1044	28 780	319 214	0.16	8.4	0.08	1 to 141
			0-10 vs ≥11	80	1168	12 700	335 294	0.06	9.4	0.04	1 to 159
			0-11 vs ≥12	34	1214	4 305	343 689	0.03	9.7	0.01	1 to 127
CAIDE Score	0 to 17	0.71 (0.70 to 0.73)									
(APOE supplemented)			0-5 vs ≥6	981	58	198 056	93 939	0.94	0.6	0.68	1 to 202
			0-6 vs ≥7	890	149	160 184	131 811	0.86	1.4	0.55	1 to 180
			0-7 vs ≥8	792	247	127 375	164 620	0.76	2.4	0.44	1 to 161
			0-8 vs ≥9	618	421	89 521	202 474	0.59	4.1	0.31	1 to 145
			0-9 vs ≥10*	441	598	57 934	234 061	0.42	5.8	0.20	1 to 131
			0-10 vs ≥11*	315	724	36 612	255 383	0.30	7.0	0.13	1 to 116
			0-11 vs ≥12	188	851	18 670	273 325	0.18	8.2	0.06	1 to 99
			0-12 vs ≥13	120	919	9 985	282 010	0.12	8.8	0.03	1 to 83
			0-13 vs ≥14	58	981	4 375	287 620	0.06	9.4	0.01	1 to 75
BDSI	0 to 42	0.62 (0.60 to 0.64)									
			0-4 vs ≥5	722	502	139 426	198 387	0.59	4.1	0.41	1 to 193
			0-6 vs ≥7	600	624	105 940	231 873	0.49	5.1	0.31	1 to 177
			0-8 vs ≥9	597	627	104 979	232 834	0.49	5.1	0.31	1 to 176
			0-9 vs ≥10	271	953	29 166	308 647	0.22	7.8	0.09	1 to 108
			0-14 vs ≥15	214	1010	24 125	313 688	0.17	8.3	0.07	1 to 113
			0-15 vs ≥16	79	1145	4 231	333 582	0.06	9.4	0.01	1 to 54
			0-21 vs ≥22*	27	1197	828	336 985	0.02	9.8	0.002	1 to 31
ANU-ADRI	-18 to 26	0.56 (0.54 to 0.58)									
			≤-11 vs ≥-10	770	139	218 968	46 719	0.85	1.5	0.82	1 to 284
			≤-6 vs ≥-5	557	352	144 540	121 147	0.61	3.9	0.54	1 to 259
			≤-4 vs ≥-3	455	454	109 229	156 458	0.50	5.0	0.41	1 to 240
			≤-3 vs ≥-2	402	507	92 064	173 623	0.44	5.6	0.35	1 to 229
			≤0 vs ≥1	260	649	50 222	215 465	0.29	7.1	0.19	1 to 193
			≤4 vs ≥5	124	785	17 941	247 746	0.14	8.6	0.07	1 to 145
			≤5 vs ≥6	89	820	13 591	252 096	0.10	9.0	0.05	1 to 153
			≤6 vs ≥7	66	843	9 969	255 718	0.07	9.3	0.04	1 to 151
			≤9 vs ≥10	34	875	3 723	261 964	0.04	9.6	0.01	1 to 110
Age only	38 to 64	0.75 (0.74 to 0.77)									
			≤49 vs ≥50	1359	58	266 856	109 407	0.96	0.4	0.71	1 to 196
			≤54 vs ≥55	1235	182	195 959	180 304	0.87	1.3	0.52	1 to 159
			≤59 vs ≥60	948	469	111 954	264 309	0.67	3.3	0.30	1 to 118
			≤63 vs ≥64	308	1109	20 470	355 793	0.22	7.8	0.05	1 to 66

Abbreviations: ANU-ADRI, the Australian National University Alzheimer's Disease Risk Index; BDSI, the Brief Dementia Screening Indicator; CAIDE, the Cardiovascular Risk Factors, Aging, and Incidence of Dementia Score; CI, confidence interval.
*Cut offs recommended in literature.

© 2023 Kivimäki M et al. *JAMA Network Open.*

eTable 5. Capacity of Dementia Risk Scores and Age Only to Estimate 10-Year Dementia Risk Among Participants Aged 65 Years or Older

Risk score	Observed range	C-index (95% CI)	Alternative cut offs	Dementia: Test pos	Dementia: Test neg	No dementia: Test pos	No dementia: Test neg	Detection rate	Number missed per 10 cases	False positive rate	Ratio true-to-false positives
CAIDE Score	4 to 14	0.53 (0.51 to 0.54)									
(Clinical version)			0-5 vs ≥6	1528	202	66 893	10 546	0.88	1.2	0.86	1 to 44
			0-6 vs ≥7	1344	386	56 648	20 791	0.78	2.2	0.73	1 to 42
			0-7 vs ≥8	973	757	40 299	37 140	0.56	4.4	0.52	1 to 41
			0-8 vs ≥9*	640	1090	26 727	50 712	0.37	6.3	0.35	1 to 42
			0-9 vs ≥10*	356	1374	14 197	63 242	0.21	7.9	0.18	1 to 40
			0-10 vs ≥11	188	1542	6 704	70 735	0.11	8.9	0.09	1 to 36
			0-11 vs ≥12	80	1650	2 409	75 030	0.05	9.5	0.03	1 to 30
CAIDE Score	5 to 17	0.60 (0.59 to 0.62)									
(APOE supplemented)			0-5 vs ≥6	1445	18	62 690	1 929	0.99	0.1	0.97	1 to 43
			0-6 vs ≥7	1384	79	57 169	7 450	0.95	0.5	0.88	1 to 41
			0-7 vs ≥8	1289	174	50 348	14 271	0.88	1.2	0.78	1 to 39
			0-8 vs ≥9	1086	377	39 002	25 617	0.74	2.6	0.60	1 to 36
			0-9 vs ≥10*	866	597	28 392	36 227	0.59	4.1	0.44	1 to 33
			0-10 vs ≥11*	642	821	19 971	44 648	0.44	5.6	0.31	1 to 31
			0-11 vs ≥12	405	1058	11 309	53 310	0.28	7.2	0.18	1 to 28
			0-12 vs ≥13	279	1184	6 502	58 117	0.19	8.1	0.10	1 to 23
			0-13 vs ≥14	139	1324	3 047	61 572	0.10	9.0	0.05	1 to 22
BDSI	0 to 38	0.61 (0.59 to 0.62)									
			0-4 vs ≥5	1188	543	42 519	34 281	0.69	3.1	0.55	1 to 36
			0-6 vs ≥7	1163	568	41 156	35 644	0.67	3.3	0.54	1 to 35
			0-8 vs ≥9	936	795	31 059	45 741	0.54	4.6	0.40	1 to 33
			0-9 vs ≥10	327	1404	7 338	69 462	0.19	8.1	0.10	1 to 22
			0-14 vs ≥15	283	1448	5 795	71 005	0.16	8.4	0.08	1 to 20
			0-15 vs ≥16	100	1631	1 438	75 362	0.06	9.4	0.02	1 to 14
			0-21 vs ≥22*	69	1662	787	76 013	0.04	9.6	0.01	1 to 11
ANU-ADRI	-17 to 28	0.57 (0.55 to 0.58)									
			≤-11 vs ≥-10	1147	123	52 360	6 828	0.90	1.0	0.88	1 to 46
			≤-6 vs ≥-5	883	387	36 522	22 666	0.70	3.0	0.62	1 to 41
			≤-4 vs ≥-3	819	451	33 233	25 955	0.64	3.6	0.56	1 to 41
			≤-3 vs ≥-2	461	809	15 802	43 386	0.36	6.4	0.27	1 to 34
			≤0 vs ≥1	251	1019	6 738	52 450	0.20	8.0	0.11	1 to 27
			≤4 vs ≥5	204	1066	5 206	53 982	0.16	8.4	0.09	1 to 26
			≤5 vs ≥6	163	1107	3 919	55 269	0.13	8.7	0.07	1 to 24
			≤6 vs ≥7	83	1187	1 568	57 620	0.07	9.3	0.03	1 to 19
			≤9 vs ≥10	14	1256	271	58 917	0.01	9.9	0.005	1 to 19
Age only	65 to 73	0.58 (0.56 to 0.59)									
			≤65 vs ≥66	1693	311	66 095	20 150	0.84	1.6	0.77	1 to 39
			≤66 vs ≥67	1330	674	47 825	38 420	0.66	3.4	0.55	1 to 36
			≤67 vs ≥68	958	1046	31 381	54 864	0.48	5.2	0.36	1 to 33
			≤68 vs ≥69	549	1455	16 401	69 844	0.27	7.3	0.19	1 to 30
			≤69 vs ≥70	85	1919	2 173	84 072	0.04	9.6	0.03	1 to 26

Abbreviations: ANU-ADRI, the Australian National University Alzheimer's Disease Risk Index; BDSI, the Brief Dementia Screening Indicator; CAIDE, the Cardiovascular Risk Factors, Aging, and Incidence of Dementia Score; CI, confidence interval.
*Cut offs recommended in literature.

© 2023 Kivimäki M et al. *JAMA Network Open.*

eTable 6. Capacity of Dementia Risk Scores and Age Only to Estimate 10-Year Dementia Risk in All Participants Including Those with Follow-up Less Than 365 Days

Risk score	Observed range	C-index (95% CI)	Alternative cut offs	Dementia		No dementia		Detection rate	Number missed per 10 cases	False positive rate	Ratio true-to-false positives
				Test pos	Test neg	Test pos	Test neg				
CAIDE Score	0 to 14	0.66 (0.65 to 0.67)									
(Clinical version)			0-5 vs ≥6	2569	466	269 987	165 110	0.85	1.5	0.62	1 to 105
			0-6 vs ≥7	2205	830	209 036	226 061	0.73	2.7	0.48	1 to 95
			0-7 vs ≥8	1587	1448	140 098	294 999	0.52	4.8	0.32	1 to 88
			0-8 vs ≥9*	1030	2005	87 626	347 471	0.34	6.6	0.20	1 to 85
			0-9 vs ≥10*	573	2462	44 178	390 919	0.19	8.1	0.10	1 to 77
			0-10 vs ≥11	273	2762	19 930	415 167	0.09	9.1	0.05	1 to 73
			0-11 vs ≥12	117	2918	6 914	428 183	0.04	9.6	0.02	1 to 59
CAIDE Score	0 to 17	0.72 (0.72 to 0.73)									
(APOE supplemented)			0-5 vs ≥6	2478	78	266 864	97 722	0.97	0.3	0.73	1 to 108
			0-6 vs ≥7	2326	230	222 564	142 022	0.91	0.9	0.61	1 to 96
			0-7 vs ≥8	2127	429	182 065	182 521	0.83	1.7	0.50	1 to 86
			0-8 vs ≥9	1737	819	131 763	232 823	0.68	3.2	0.36	1 to 76
			0-9 vs ≥10*	1334	1222	88 529	276 057	0.52	4.8	0.24	1 to 66
			0-10 vs ≥11*	978	1578	58 017	306 569	0.38	6.2	0.16	1 to 59
			0-11 vs ≥12	607	1949	30 739	333 847	0.24	7.6	0.08	1 to 51
			0-12 vs ≥13	410	2146	16 919	347 667	0.16	8.4	0.05	1 to 41
			0-13 vs ≥14	206	2350	7 604	356 982	0.08	9.2	0.02	1 to 37
BDSI	0 to 42	0.68 (0.67 to 0.69)									
			0-4 vs ≥5	1943	1068	186 533	237 514	0.65	3.5	0.44	1 to 96
			0-6 vs ≥7	1745	1266	149 519	274 528	0.58	4.2	0.35	1 to 86
			0-8 vs ≥9	1666	1345	146 566	277 481	0.55	4.5	0.35	1 to 88
			0-9 vs ≥10	1230	1781	61 925	362 122	0.41	5.9	0.15	1 to 50
			0-14 vs ≥15	557	2454	32 450	391 597	0.18	8.2	0.08	1 to 58
			0-15 vs ≥16	372	2639	10 392	413 655	0.12	8.8	0.02	1 to 28
			0-21 vs ≥22*	102	2909	1 696	422 351	0.03	9.7	0.004	1 to 17
ANU-ADRI	-18 to 28	0.59 (0.57 to 0.60)									
			≤-11 vs ≥-10	1951	268	277 590	54 633	0.88	1.2	0.84	1 to 142
			≤-6 vs ≥-5	1465	754	185 405	146 818	0.66	3.4	0.56	1 to 127
			≤-4 vs ≥-3	1213	1006	142 020	190 203	0.55	4.5	0.43	1 to 117
			≤-3 vs ≥-2	1087	1132	120 791	211 432	0.49	5.1	0.36	1 to 111
			≤0 vs ≥1	735	1484	67 729	264 494	0.33	6.7	0.20	1 to 92
			≤4 vs ≥5	382	1837	25 360	306 863	0.17	8.3	0.08	1 to 66
			≤5 vs ≥6	297	1922	19 302	312 921	0.13	8.7	0.06	1 to 65
			≤6 vs ≥7	232	1987	14 256	317 967	0.10	9.0	0.04	1 to 61
			≤9 vs ≥10	119	2100	5 431	326 792	0.05	9.5	0.02	1 to 46
Age only	38 to 73	0.79 (0.79 to 0.80)									
			≤49 vs ≥50	3424	63	361 300	111 650	0.98	0.2	0.76	1 to 106
			≤54 vs ≥55	3296	191	288 850	184 100	0.95	0.5	0.61	1 to 88
			≤59 vs ≥60	3003	484	202 869	270 081	0.86	1.4	0.43	1 to 68
			≤63 vs ≥64	2350	1137	109 233	363 717	0.67	3.3	0.23	1 to 46
			≤64 vs ≥65	2039	1448	88 283	384 667	0.58	4.2	0.19	1 to 43
			≤65 vs ≥66	1720	1767	67 644	405 306	0.49	5.1	0.14	1 to 39
			≤66 vs ≥67	1352	2135	48 951	423 999	0.39	6.1	0.10	1 to 36
			≤67 vs ≥68	974	2513	32 061	440 889	0.28	7.2	0.07	1 to 33
			≤68 vs ≥69	557	2930	16 730	456 220	0.16	8.4	0.04	1 to 30
			≤69 vs ≥70	85	3402	2 201	470 749	0.02	9.8	0.005	1 to 26

Abbreviations: ANU-ADRI, the Australian National University Alzheimer's Disease Risk Index; BDSI, the Brief Dementia Screening Indicator; CAIDE, the Cardiovascular Risk Factors, Aging, and Incidence of Dementia Score; CI, confidence interval.
*Cut offs recommended in literature.

© 2023 Kivimäki M et al. *JAMA Network Open.*

eTable 7. Performance of Dementia Risk Scores and Age Only to Estimate 10-Year Dementia Risk at Age 65 (+1) Years

Risk score	Observed range	C-index (95% CI)	Alternative cut offs	Dementia		No dementia		Detection rate	Number missed per 10 cases	False positive rate	Ratio true-to-false positives
				Test pos	Test neg	Test pos	Test neg				
CAIDE Score	4 to 14	0.52 (0.50 to 0.54)									
(Clinical version)			0-5 vs ≥6	745	114	45 307	7 966	0.87	1.3	0.85	1 to 61
			0-6 vs ≥7	644	215	37 852	15 421	0.75	2.5	0.71	1 to 59
			0-7 vs ≥8	466	393	26 625	26 648	0.54	4.6	0.50	1 to 57
			0-8 vs ≥9*	289	570	17 443	35 830	0.34	6.6	0.33	1 to 60
			0-9 vs ≥10*	160	699	9 256	44 017	0.19	8.1	0.17	1 to 58
			0-10 vs ≥11	70	789	4 323	48 950	0.08	9.2	0.08	1 to 62
			0-11 vs ≥12	35	824	1 623	51 650	0.04	9.6	0.03	1 to 46
CAIDE Score	5 to 17	0.60 (0.58 to 0.62)									
(APOE supplemented)			0-5 vs ≥6	706	7	42 650	1 521	0.99	0.1	0.97	1 to 60
			0-6 vs ≥7	670	43	38 536	5 635	0.94	0.6	0.87	1 to 58
			0-7 vs ≥8	628	85	33 519	10 652	0.88	1.2	0.76	1 to 53
			0-8 vs ≥9	530	183	25 718	18 453	0.74	2.6	0.58	1 to 49
			0-9 vs ≥10*	401	312	18 464	25 707	0.56	4.4	0.42	1 to 46
			0-10 vs ≥11*	281	432	12 757	31 414	0.39	6.1	0.29	1 to 45
			0-11 vs ≥12	166	547	7 023	37 148	0.23	7.7	0.16	1 to 42
			0-12 vs ≥13	109	604	4 029	40 142	0.15	8.5	0.09	1 to 37
			0-13 vs ≥14	54	659	1 946	42 225	0.08	9.2	0.04	1 to 36
BDSI	0 to 34	0.59 (0.57 to 0.60)									
			0-4 vs ≥5	531	336	26 265	26 425	0.61	3.9	0.50	1 to 49
			0-6 vs ≥7	479	388	24 494	28 196	0.55	4.5	0.46	1 to 49
			0-8 vs ≥9	450	417	23 715	28 975	0.52	4.8	0.45	1 to 53
			0-9 vs ≥10	284	583	11 371	41 319	0.33	6.7	0.22	1 to 40
			0-14 vs ≥15	134	733	3 879	48 811	0.15	8.5	0.07	1 to 29
			0-15 vs ≥16	81	786	1 832	50 858	0.09	9.1	0.03	1 to 23
			0-21 vs ≥22*	14	853	198	52 492	0.02	9.8	0.004	1 to 14
ANU-ADRI	-18 to 22	0.57 (0.55 to 0.60)									
			≤-11 vs ≥-10	579	76	34 118	6 922	0.88	1.2	0.83	1 to 59
			≤-6 vs ≥-5	417	238	22 269	18 771	0.64	3.6	0.54	1 to 53
			≤-4 vs ≥-3	337	318	17 175	23 865	0.51	4.9	0.42	1 to 51
			≤-3 vs ≥-2	298	357	14 748	26 292	0.45	5.5	0.36	1 to 49
			≤0 vs ≥1	195	460	8 333	32 707	0.30	7.0	0.20	1 to 43
			≤4 vs ≥5	97	558	3 064	37 976	0.15	8.5	0.07	1 to 32
			≤5 vs ≥6	74	581	2 321	38 719	0.11	8.9	0.06	1 to 31
			≤6 vs ≥7	52	603	1 664	39 376	0.08	9.2	0.04	1 to 32
			≤9 vs ≥10	25	630	538	40 502	0.038	9.6	0.01	1 to 22

Abbreviations: ANU-ADRI, the Australian National University Alzheimer's Disease Risk Index; BDSI, the Brief Dementia Screening Indicator; CAIDE Score, the Cardiovascular Risk Factors, Aging, and Incidence of Dementia Score; CI, condidence interval.
*Cut offs recommended in literature.

© 2023 Kivimäki M et al. *JAMA Network Open.*

eTable 8. Capacity of Dementia Risk Scores and Age Only to Estimate 20-Year Dementia Risk in the Whitehall II Study

Risk score	Observed range	C-index (95% CI)	Alternative cut offs	Dementia		No dementia		Detection rate	Number missed per 10 cases	False positive rate	Ratio true-to-false positives
				Test pos	Test neg	Test pos	Test neg				
**20-YEAR DEMENTIA RISK**											
CAIDE Score	0 to 13	0.65 (0.61 to 0.68)									
(Clinical version)			0-5 vs 6+	165	37	2,706	1,957	0.82	1.8	0.58	1 to 16
			0-6 vs 7+	111	91	1,771	2,892	0.55	4.5	0.38	1 to 16
			0-7 vs 8+	75	127	1,001	3,662	0.37	6.3	0.21	1 to 13
			0-8 vs 9+*	39	163	488	4,175	0.19	8.1	0.10	1 to 13
			0-9 vs 10+*	19	183	224	4,439	0.09	9.1	0.05	1 to 12
			0-10 vs 11+	11	191	84	4,579	0.05	9.5	0.02	1 to 8
CAIDE Score	0 to 15	0.74 (0.70 to 0.78)									
(APOE supplemented)			0-5 vs 6+	136	9	2,608	1,032	0.94	0.6	0.72	1 to 19
			0-6 vs 7+	126	19	2,035	1,605	0.87	1.3	0.56	1 to 16
			0-7 vs 8+	105	40	1,286	2,354	0.72	2.8	0.35	1 to 12
			0-8 vs 9+	87	58	837	2,803	0.60	4.0	0.23	1 to 10
			0-9 vs 10+*	52	93	446	3,194	0.36	6.4	0.12	1 to 9
			0-10 vs 11+*	34	111	233	3,407	0.23	7.7	0.06	1 to 7
			0-11 vs 12+	15	130	111	3,529	0.10	9.0	0.03	1 to 7
			0-12 vs 13+	7	138	39	3,601	0.05	9.5	0.01	1 to 6
Age only	45 to 69	0.79 (0.77 to 0.82)									
			≤49 vs 50+	200	2	3,664	999	0.99	0.1	0.79	1 to 18
			≤54 vs 55+	183	19	2,223	2,440	0.91	0.9	0.48	1 to 12
			≤59 vs 60+	145	57	1,250	3,413	0.72	2.8	0.27	1 to 9
			≤63 vs 64+	79	123	513	4,150	0.39	6.1	0.11	1 to 6
			≤64 vs 65+	40	162	336	4,327	0.20	8.0	0.07	1 to 8
			≤65 vs 66+	18	184	158	4,505	0.09	9.1	0.03	1 to 9
			≤66 vs 67+	9	193	34	4,629	0.04	9.6	0.01	1 to 4

Abbreviations: ANU-ADRI, the Australian National University Alzheimer's Disease Risk Index; BDSI, the Brief Dementia Screening Indicator; CAIDE, the Cardiovascular Risk Factors, Aging, and Incidence of Dementia Score; CI, confidence interval; NA, not available, because there were no true positives in the sample.
*Cut offs recommended in literature.

© 2023 Kivimäki M et al. *JAMA Network Open.*

Statistical code

```
version 17

**** UK BIOBANK ****

capture log close
log using "Analysis.log", append

use "UKB_dementia_risk_scores_12122022.dta", clear
merge 1:1 n_eid using "ethnic background.dta"
drop if _merge==2
drop _merge

tab ethnic
gen non_white = !inlist(ethnic,1,99)
tab non_white ethnic, mis

*Put dementia to missing if dementia diagnosis was recorded after the end of the follow-up
count if dementia_date > date_exit & dementia_date<.
replace dementia_date = . if dementia_date > date_exit & dementia_date<.
replace dementia = 0 if dementia_date==.
replace dementia_code = "" if dementia_date==.

*Flowchart

*Attended baseline assessment
*503317 (from Fry A et al. Am J Epidemiol. 2017 Nov 1;186(9):1026-1034. PMID: 28641372)
*Available in our data
count
*502388
di 503317-502388
*929
di 929/503310
*0.2%
count if date_recruit!=.
*502,388

*Missing data for each risk score:

*CAIDE1:
gen full_data_caide_1 = 1
foreach var in date_recruit age_caide_1 education_caide_1 sex_caide_1 sbp_caide_1 bmi_caide_1 tot_chol_caide_1
phyact_caide_1 {
	replace full_data_caide_1 = 0 if `var'==.
}
tab full_data_caide_1

*CAIDE2:
gen full_data_caide_2 = 1
foreach var in date_recruit age_caide_2 education_caide_2 sex_caide_2 sbp_caide_2 bmi_caide_2 tot_chol_caide_2
phyact_caide_2 apoe_caide_2 {
	replace full_data_caide_2 = 0 if `var'==.
}
tab full_data_caide_2

*BRIEF:
gen full_data_brief = 1
```

© 2023 Kivimäki M et al. *JAMA Network Open.*

```
foreach var in date_recruit age_brief education_brief bmi_brief diabetes_brief stroke_brief help_brief depression_brief
{
	replace full_data_brief = 0 if `var'==.
}
tab full_data_brief

*anu_adri:
gen full_data_anu_adri = 1
foreach var in date_recruit age_anu_adri education_anu_adri bmi_anu_adri diabetes_anu_adri depression_anu_adri
tot_chol_anu_adri tbi_anu_adri smoker_anu_adri alcohol_anu_adri social_anu_adri phyact_anu_adri
cogn_act_anu_adri fish_per_week_anu_adri pesticides_ever_anu_adri  {
	replace full_data_anu_adri = 0 if `var'==.
}
tab full_data_anu_adri

*Full data for at least one risk score:
egen full_data_any_score = rowmax(full_data_caide_1 full_data_caide_2 full_data_brief full_data_anu_adri)
tab full_data_any_score
*476525

di 502388-476525
*25863
di (502388-476525)/(503317)
*5.2%

*Total exclusions at this point
di 929 + 25863
*26792
di 26792/503317
*5.3%

*Aged <40 or >70
gen out_of_age = age_recruit<40 | age_recruit>70
tab out_of_age
*17
di 17/503317
*0.0%
*Do not drop these at this moment

*Dementia at baseline
gen dementia_baseline = dementia_date<=date_recruit & date_recruit!=.
tab dementia_baseline
tab dementia_baseline if full_data_any_score==1
*88 (0.02%)

*Follow up less than 365 days
gen short_fu = (date_exit - date_recruit)<365
tab short_fu
tab short_fu if dementia_baseline==0 & full_data_any_score==1
*978 (0.2%)

*Cause of short follow-up
gen short_dem = dementia_baseline==0 & full_data_any_score==1 & short_fu==1 & dementia==1
tab short_dem if dementia_baseline==0 & full_data_any_score==1
gen short_death = dementia_baseline==0 & full_data_any_score==1 & short_fu==1 & dementia==0 & (date_death -
date_recruit)<365
tab short_death if dementia_baseline==0 & full_data_any_score==1
gen short_lost = dementia_baseline==0 & full_data_any_score==1 & short_fu==1 & dementia==0 & (date_lost -
date_recruit)<365
tab short_lost if dementia_baseline==0 & full_data_any_score==1
```

© 2023 Kivimäki M et al. *JAMA Network Open.*

```
*dementia at baseline 40
*died too soon 869
*lost to follow-up too soon 69
di 40+869+69

*Theoretical follow-up less than 10-years
capture drop theor_fu
gen theor_fu = (date_end_of_fu - date_recruit)/365.25
capture drop theor_fu_10
egen theor_fu_10 = cut(theor_fu), at(0,10,100)
tab theor_fu_10
tab theor_fu_10 if dementia_baseline==0 & short_fu==0 & full_data_any_score==1
*9530 (2.0%)

di 88 + 978 + 9530
*10596

di 10596/476525
*2.2%

*10596 (2.2%) excluded

*GENERATE VARIABLES FOR ANALYTIC SAMPLES
gen sample = full_data_any_score==1 & theor_fu_10==10 & short_fu==0 & dementia_baseline==0
tab sample
*465929 in sample

foreach score in caide_1 caide_2 brief anu_adri {
capture drop sample_`score'
gen sample_`score' = full_data_`score'==1 & dementia_baseline==0 & short_fu==0 & theor_fu>=10
tab sample_`score'
}
gen sample_age_recruit = sample

*SAMPLE FOR SENSITIVITY ANALYSIS THAT DOES NOT EXCLUDE SHORT FOLLOW UP
gen sample_sens = full_data_any_score==1 & dementia_baseline==0
tab sample_sens
*476437 in sample

foreach score in caide_1 caide_2 brief anu_adri {
capture drop sample_sens_`score'
gen sample_sens_`score' = full_data_`score'==1 & dementia_baseline==0
tab sample_sens_`score'
}
gen sample_sens_age_recruit = sample_sens


*GENERATE DEMENTIA RISK SCORES
gen caide_1 = age_caide_1 + education_caide_1 + sex_caide_1 + sbp_caide_1 + bmi_caide_1 + tot_chol_caide_1 +
phyact_caide_1
tab sample_caide_1
sum caide_1 if sample_caide_1==1

gen caide_2 = age_caide_2 + education_caide_2 + sex_caide_2 + sbp_caide_2 + bmi_caide_2 + tot_chol_caide_2 +
phyact_caide_2 + apoe_caide_2
tab sample_caide_2
sum caide_2 if sample_caide_2==1

gen brief = age_brief + education_brief + bmi_brief + diabetes_brief + stroke_brief + help_brief + depression_brief
tab sample_brief
```

© 2023 Kivimäki M et al. *JAMA Network Open.*

```
sum brief if sample_brief==1

gen anu_adri = age_anu_adri + education_anu_adri + bmi_anu_adri + diabetes_anu_adri + depression_anu_adri +
tot_chol_anu_adri + tbi_anu_adri + smoker_anu_adri + alcohol_anu_adri + social_anu_adri + phyact_anu_adri +
cogn_act_anu_adri + fish_per_week_anu_adri + pesticides_ever_anu_adri
tab sample_anu_adri
sum anu_adri if sample_anu_adri==1

*CHECK ITEMS OF DEMENTIA RISK SCORES

*CAIDE 1:
tab age_caide_1
bysort age_caide_1: sum age_recruit

tab education_caide_1
bysort education_caide_1: sum education_years

tab sex_caide_1
bysort sex_caide_1: sum sex

tab sbp_caide_1
bysort sbp_caide_1: sum sbp

tab bmi_caide_1
bysort bmi_caide_1: sum bmi

tab tot_chol_caide_1
bysort tot_chol_caide_1: sum tot_chol

tab phyact_caide_1
bysort phyact_caide_1: sum inactive
tab phyact_caide_1 vig
*All correct

*CAIDE 2:
tab age_caide_2
bysort age_caide_2: sum age_recruit

tab education_caide_2
bysort education_caide_2: sum education_years

tab sex_caide_2
bysort sex_caide_2: sum sex

tab sbp_caide_2
bysort sbp_caide_2: sum sbp

tab bmi_caide_2
bysort bmi_caide_2: sum bmi

tab tot_chol_caide_2
bysort tot_chol_caide_2: sum tot_chol

tab phyact_caide_2
bysort phyact_caide_2: sum inactive
tab phyact_caide_2 vig

tab apoe_caide_2
tab apoe_caide_2 apoe
*All correct
```

© 2023 Kivimäki M et al. *JAMA Network Open.*

```
*BRIEF
tab age_brief
bysort age_brief: sum age_recruit

tab education_brief
bysort education_brief: sum education_years

tab bmi_brief
bysort bmi_brief: sum bmi

tab diabetes_brief
tab diabetes_brief diabetes

tab stroke_brief
tab stroke_brief stroke

tab help_brief

tab depression_brief
*All correct

*ANU-ADRI
tab age_anu_adri if sex==1
tab age_anu_adri if sex==0
bysort sex age_anu_adri: sum age_recruit

tab education_anu_adri
bysort education_anu_adri: sum education_years

tab bmi_anu_adri
tab bmi_anu_adri if age_recruit >=60
bysort bmi_anu_adri: sum bmi
bysort bmi_anu_adri: sum bmi if age_recruit <60
bysort bmi_anu_adri: sum bmi if age_recruit >=60

tab diabetes_anu_adri
tab diabetes_anu_adri diabetes

tab depression_anu_adri

tab tot_chol_anu_adri
tab tot_chol_anu_adri if age_recruit>=60
bysort tot_chol_anu_adri: sum tot_chol if age_recruit<60
bysort tot_chol_anu_adri: sum tot_chol if age_recruit>=60

tab tbi_anu_adri
tab tbi_anu_adri tbi

tab smoker_anu_adri
tab smoker_anu_adri smoker

tab alcohol_anu_adri
tab alcohol_anu_adri abstainer

tab social_anu_adri
tab social_anu_adri social_saczynski_cut

tab phyact_anu_adri
tab phyact_anu_adri ipaq
```

© 2023 Kivimäki M et al. *JAMA Network Open.*

```
tab cogn_act_anu_adri
tab cogn_act_anu_adri cogn_act

tab fish_per_week_anu_adri
tab fish_per_week_anu_adri fish_per_week

tab pesticides_ever_anu_adri
tab pesticides_ever_anu_adri pesticides_ever
*All correct


**** 10-year dementia risk among all****
capture drop date_exit_10
capture drop dementia_10
gen date_exit_10 = date_recruit + round(10*365.25)
stset date_exit, failure(dementia) id(n_eid) origin(date_recruit) scale(365.25) exit(date_exit_10)
gen dementia_10 = _d==1 if date_recruit!=.
tab dementia_10
*Put to missing those who had dementia at baseline, less than 365 days of follow up or less than ten years of theoretical follow up:
replace dementia_10 = . if dementia_baseline==1
replace dementia_10 = . if short_fu==1
replace dementia_10 = . if theor_fu_10==0
*Indicators for the availability of the scores
capture gen caide_1_available = 1 if caide_1!=.
capture gen caide_2_available = 1 if caide_2!=.
capture gen brief_available = 1 if brief!=.
capture gen anu_adri_available = 1 if anu_adri!=.

*Missing table with column percentage
	baselinetable ///
		age_recruit(cts novarlabel afterhead("Age at entry, years")) ///
		sex(novarlabel afterhead("Sex")) ///
		non_white(novarlabel afterhead("Non-white ethnicity")) ///
		caide_1_available (novarlabel afterhead("CAIDE score (clinical version) available")) ///
		caide_1(cts novarlabel afterhead("CAIDE score (clinical version)")) ///
		age_caide_1(novarlabel afterhead("Age, CAIDE (clinical version)")) ///
		sex_caide_1 (novarlabel afterhead("Sex, CAIDE (clinical version)")) ///
		education_caide_1 (novarlabel afterhead("Education, CAIDE (clinical version)")) ///
		sbp_caide_1 (novarlabel afterhead("Systolic blood pressure, CAIDE (clinical version)")) ///
		bmi_caide_1 (novarlabel afterhead("Body mass index, CAIDE (clinical version)")) ///
		tot_chol_caide_1 (novarlabel afterhead("Total cholesterol, CAIDE (clinical version)")) ///
		phyact_caide_1 (novarlabel afterhead("Physical activity, CAIDE (clinical version)")) ///
		caide_2_available (novarlabel afterhead("CAIDE score (APOE supplemented) available")) ///
		caide_2(cts novarlabel afterhead("CAIDE score (APOE supplemented)")) ///
		age_caide_2(novarlabel afterhead("Age, CAIDE (APOE supplemented)")) ///
		sex_caide_2 (novarlabel afterhead("Sex, CAIDE (APOE supplemented)")) ///
		education_caide_2 (novarlabel afterhead("Education, CAIDE (APOE supplemented)")) ///
		sbp_caide_2 (novarlabel afterhead("Systolic blood pressure, CAIDE (APOE supplemented)")) ///
		bmi_caide_2 (novarlabel afterhead("Body mass index, CAIDE (APOE supplemented)")) ///
		tot_chol_caide_2 (novarlabel afterhead("Total cholesterol, CAIDE (APOE supplemented)")) ///
		phyact_caide_2 (novarlabel afterhead("Physical activity, CAIDE (APOE supplemented)")) ///
		apoe_caide_2 (novarlabel afterhead("APOE4 carrier, CAIDE (APOE supplemented)")) ///
		brief_available (novarlabel afterhead("Brief score available")) ///
		brief(cts novarlabel afterhead("Brief score")) ///
		age_brief (novarlabel afterhead("Age, Brief")) ///
		education_brief (novarlabel afterhead("Education, Brief")) ///
		bmi_brief (novarlabel afterhead("Body mass index, Brief")) ///
		diabetes_brief (novarlabel afterhead("Diabetes, Brief")) ///
```

© 2023 Kivimäki M et al. *JAMA Network Open.*

```
		stroke_brief (novarlabel afterhead("History of stroke, Brief")) ///
		help_brief (novarlabel afterhead("Needs help, Brief")) ///
		depression_brief (novarlabel afterhead("Depressive symptoms, Brief")) ///
		anu_adri_available (novarlabel afterhead("ANU-ADRI score available")) ///
		anu_adri(cts novarlabel afterhead("ANU-ADRI score")) ///
		age_anu_adri (novarlabel afterhead("Age, ANU-ADRI")) ///
		education_anu_adri (novarlabel afterhead("Education, ANU-ADRI")) ///
		bmi_anu_adri (novarlabel afterhead("Body mass index, ANU-ADRI")) ///
		diabetes_anu_adri (novarlabel afterhead("Diabetes, ANU-ADRI")) ///
		depression_anu_adri (novarlabel afterhead("Depressive symptoms, ANU-ADRI")) ///
		tot_chol_anu_adri (novarlabel afterhead("Total cholesterol, ANU-ADRI")) ///
		tbi_anu_adri (novarlabel afterhead("History of traumatic brain injury, ANU-ADRI")) ///
		smoker_anu_adri (novarlabel afterhead("Smoking status, ANU-ADRI")) ///
		alcohol_anu_adri (novarlabel afterhead("Alcohol drinking, ANU-ADRI")) ///
		social_anu_adri (novarlabel afterhead("Social engagement, ANU-ADRI")) ///
		phyact_anu_adri (novarlabel afterhead("Physical activity, ANU-ADRI")) ///
		cogn_act_anu_adri (novarlabel afterhead("Cognitive activity, ANU-ADRI")) ///
		fish_per_week_anu_adri (novarlabel afterhead("Fish intake, ANU-ADRI")) ///
		pesticides_ever_anu_adri (novarlabel afterhead("Pesticide exposure, ANU-ADRI")) ///
		, by(dementia_10, total) ctsvartab(mean (sd)) catvartab(# (%\%)) missing ///
		exportexcel(Table_data_vs_missing_12122022, cell(A6) replace)


*Simple missing table with column percentage
capture label define missing 0 "Available" 1 "Missing"
foreach var in age_recruit sex ethnic education_years sbp_caide_1 bmi_caide_1 tot_chol_caide_1 vig2 apoe_caide_2
diabetes stroke help depression_brief tbi smoker alcohol_anu_adri social_saczynski_cut ipaq_012 cogn_act
fish_per_week pesticides_ever {
   di ""
	di "`var'"
	tab `var', mis
	capture drop missing_`var'
	gen missing_`var' = inlist(`var',.)
	if "`var'" == "ethnic" {
	   replace missing_`var' = 1 if `var'==99
	}
	label values missing_`var' missing
	tab missing_`var'
}

	baselinetable ///
		missing_age_recruit(novarlabel afterhead("Age at entry, years")) ///
		missing_sex(novarlabel afterhead("Sex")) ///
		missing_ethnic(novarlabel afterhead("Ethnicity")) ///
		missing_education_years (novarlabel afterhead("Education, years")) ///
		missing_sbp_caide_1 (novarlabel afterhead("Systolic blood pressure")) ///
		missing_bmi_caide_1 (novarlabel afterhead("Body mass index")) ///
		missing_tot_chol_caide_1 (novarlabel afterhead("Total cholesterol")) ///
		missing_vig2 (novarlabel afterhead("Physical activity, times per week")) ///
		missing_ipaq_012 (novarlabel afterhead("Physical activity, IPAQ group")) ///
		missing_apoe_caide_2 (novarlabel afterhead("APOE4 carrier")) ///
		missing_diabetes (novarlabel afterhead("Diabetes mellitus")) ///
		missing_stroke (novarlabel afterhead("History of stroke")) ///
		missing_help (novarlabel afterhead("Needs help")) ///
		missing_depression_brief (novarlabel afterhead("Depressive symptoms")) ///
		missing_tbi (novarlabel afterhead("History of traumatic brain injury")) ///
		missing_smoker (novarlabel afterhead("Smoking status")) ///
		missing_alcohol (novarlabel afterhead("Alcohol drinking")) ///
		missing_social (novarlabel afterhead("Social engagement")) ///
		missing_cogn_act (novarlabel afterhead("Cognitive activity")) ///
```

© 2023 Kivimäki M et al. *JAMA Network Open.*

```
missing_fish_per_week (novarlabel afterhead("Fish intake")) ///
missing_pesticides_ever (novarlabel afterhead("Occupational pesticide exposure")) ///
, catvartab(# (%\%)) missing ///
exportexcel(Table_missing_19122022, cell(A6) replace)

*Missing table with row percentage
baselinetable ///
age_recruit(cts novarlabel afterhead("Age at entry, years")) ///
sex(novarlabel afterhead("Sex")) ///
non_white(novarlabel afterhead("Non-white ethnicity")) ///
caide_1_available (novarlabel afterhead("CAIDE score (clinical version) available")) ///
caide_1(cts novarlabel afterhead("CAIDE score (clinical version)")) ///
age_caide_1(novarlabel afterhead("Age, CAIDE (clinical version)")) ///
sex_caide_1 (novarlabel afterhead("Sex, CAIDE (clinical version)")) ///
education_caide_1 (novarlabel afterhead("Education, CAIDE (clinical version)")) ///
sbp_caide_1 (novarlabel afterhead("Systolic blood pressure, CAIDE (clinical version)")) ///
bmi_caide_1 (novarlabel afterhead("Body mass index, CAIDE (clinical version)")) ///
tot_chol_caide_1 (novarlabel afterhead("Total cholesterol, CAIDE (clinical version)")) ///
phyact_caide_1 (novarlabel afterhead("Physical activity, CAIDE (clinical version)")) ///
caide_2_available (novarlabel afterhead("CAIDE score (APOE supplemented) available")) ///
caide_2(cts novarlabel afterhead("CAIDE score (APOE supplemented)")) ///
age_caide_2(novarlabel afterhead("Age, CAIDE (APOE supplemented)")) ///
sex_caide_2 (novarlabel afterhead("Sex, CAIDE (APOE supplemented)")) ///
education_caide_2 (novarlabel afterhead("Education, CAIDE (APOE supplemented)")) ///
sbp_caide_2 (novarlabel afterhead("Systolic blood pressure, CAIDE (APOE supplemented)")) ///
bmi_caide_2 (novarlabel afterhead("Body mass index, CAIDE (APOE supplemented)")) ///
tot_chol_caide_2 (novarlabel afterhead("Total cholesterol, CAIDE (APOE supplemented)")) ///
phyact_caide_2 (novarlabel afterhead("Physical activity, CAIDE (APOE supplemented)")) ///
apoe_caide_2 (novarlabel afterhead("APOE4 carrier, CAIDE (APOE supplemented)")) ///
brief_available (novarlabel afterhead("Brief score available")) ///
brief(cts novarlabel afterhead("Brief score")) ///
age_brief (novarlabel afterhead("Age, Brief")) ///
education_brief (novarlabel afterhead("Education, Brief")) ///
bmi_brief (novarlabel afterhead("Body mass index, Brief")) ///
diabetes_brief (novarlabel afterhead("Diabetes, Brief")) ///
stroke_brief (novarlabel afterhead("History of stroke, Brief")) ///
help_brief (novarlabel afterhead("Needs help, Brief")) ///
depression_brief (novarlabel afterhead("Depressive symptoms, Brief")) ///
anu_adri_available (novarlabel afterhead("ANU-ADRI score available")) ///
anu_adri(cts novarlabel afterhead("ANU-ADRI score")) ///
age_anu_adri (novarlabel afterhead("Age, ANU-ADRI")) ///
education_anu_adri (novarlabel afterhead("Education, ANU-ADRI")) ///
bmi_anu_adri (novarlabel afterhead("Body mass index, ANU-ADRI")) ///
diabetes_anu_adri (novarlabel afterhead("Diabetes, ANU-ADRI")) ///
depression_anu_adri (novarlabel afterhead("Depressive symptoms, ANU-ADRI")) ///
tot_chol_anu_adri (novarlabel afterhead("Total cholesterol, ANU-ADRI")) ///
tbi_anu_adri (novarlabel afterhead("History of traumatic brain injury, ANU-ADRI")) ///
smoker_anu_adri (novarlabel afterhead("Smoking status, ANU-ADRI")) ///
alcohol_anu_adri (novarlabel afterhead("Alcohol drinking, ANU-ADRI")) ///
social_anu_adri (novarlabel afterhead("Social engagement, ANU-ADRI")) ///
phyact_anu_adri (novarlabel afterhead("Physical activity, ANU-ADRI")) ///
cogn_act_anu_adri (novarlabel afterhead("Cognitive activity, ANU-ADRI")) ///
fish_per_week_anu_adri (novarlabel afterhead("Fish intake, ANU-ADRI")) ///
pesticides_ever_anu_adri (novarlabel afterhead("Pesticide exposure, ANU-ADRI")) ///
, by(dementia_10) ctsvartab(mean (sd)) catvartab(# (r%\%)) missing ///
exportexcel(Table_data_vs_missing_row_percent_12122022, cell(A6) replace)


*Table 1 (by inclusion vs exclusion to the sample)
```

© 2023 Kivimäki M et al. *JAMA Network Open.*

```
baselinetable ///
age_recruit(cts novarlabel afterhead("Age at entry, years")) ///
sex(novarlabel afterhead("Sex")) ///
non_white(novarlabel afterhead("Non-white ethnicity")) ///
dementia_10(novarlabel afterhead("Dementia within 10 years")) ///
caide_1(cts novarlabel afterhead("CAIDE score (clinical version)")) ///
caide_2(cts novarlabel afterhead("CAIDE score (APOE supplemented)")) ///
brief(cts novarlabel afterhead("Brief score")) ///
anu_adri(cts novarlabel afterhead("ANU-ADRI score")) ///
, by(sample, total) ctsvartab(mean (sd)) missing ///
exportexcel(Table_1_by_sample, cell(A6) replace)

*p-values
tab sample
ttest age_recruit, by(sample)
tab sample sex, row chi
tab sample non_white, row chi
tab sample dementia_10, row chi


**** 10-year dementia risk in sample****
capture drop dementia_10
stset date_exit if sample==1, failure(dementia) id(n_eid) origin(date_recruit) scale(365.25) exit(date_exit_10)
gen dementia_10 = _d==1
tab dementia_10

***** TABLE 1 **************
tab ethnic, mis, if sample==1
tab ethnic non_white if sample==1

*Table 1 (by status of dementia during follow up)
sum caide_1 caide_2 brief anu_adri if sample==1
bysort dementia_10: sum caide_1 caide_2 brief anu_adri if sample==1

baselinetable ///
age_recruit(cts novarlabel afterhead("Age at entry, years")) ///
sex(novarlabel afterhead("Sex")) ///
non_white(novarlabel afterhead("Non-white ethnicity")) ///
caide_1(cts novarlabel afterhead("CAIDE score (clinical version)")) ///
caide_2(cts novarlabel afterhead("CAIDE score (APOE supplemented)")) ///
brief(cts novarlabel afterhead("Brief score")) ///
anu_adri(cts novarlabel afterhead("ANU-ADRI score")) ///
if sample==1 ///
, by(dementia_10, total) ctsvartab(mean (sd)) missing ///
exportexcel(Table_1_12122022, cell(A6) replace)

*P-values for Table 1
ttest age_recruit if sample==1, by(dementia_10)
tab sex dementia_10 if sample==1, chi
tab non_white dementia_10 if sample==1, chi
ttest caide_1 if sample==1, by(dementia_10)
ttest caide_2 if sample==1, by(dementia_10)
ttest brief if sample_brief==1, by(dementia_10)
ttest anu_adri if sample_anu_adri==1, by(dementia_10)

******* FIGURE 1 ***********

*AUC:s
foreach score in caide_1 caide_2 brief anu_adri age_recruit {
    somersd dementia_10 `score' if sample_`score'==1, transf(c)
```

© 2023 Kivimäki M et al. *JAMA Network Open.*

```
                }

        logistic dementia_10 caide_1 if sample_caide_1==1
        lroc, title("CAIDE Score (Clinical version)") ///
                ytitle("Detection rate") ///
                xtitle("False positive rate") ///
                text(0.85 0.05 "AUC: 0.66 (95% CI 0.65-0.67)", placement(east)) ///
                scheme(s1mono) ///
                xlab(0(0.25)1, nogrid) ylab(0(0.25)1, nogrid)
        graph export "ROC, caide_1.pdf", as(pdf) replace
        graph export "ROC, caide_1.emf", as(emf) replace

                logistic dementia_10 caide_2 if sample_caide_2==1
        lroc, title("CAIDE Score (APOE supplemented)") ///
                ytitle("Detection rate") ///
                xtitle("False positive rate") ///
                text(0.85 0.05 "AUC: 0.73 (95% CI 0.72-0.73)", placement(east)) ///
                scheme(s1mono) ///
                xlab(0(0.25)1, nogrid) ylab(0(0.25)1, nogrid)
        graph export "ROC, caide_2.pdf", as(pdf) replace
        graph export "ROC, caide_2.emf", as(emf) replace

        logistic dementia_10 brief if sample_brief==1
        lroc, title("BDSI") ///
                ytitle("Detection rate") ///
                xtitle("False positive rate") ///
                text(0.85 0.05 "AUC: 0.68 (95% CI 0.67-0.69)", placement(east)) ///
                scheme(s1mono) ///
                xlab(0(0.25)1, nogrid) ylab(0(0.25)1, nogrid)
        graph export "ROC, brief.pdf", as(pdf) replace
        graph export "ROC, brief.emf", as(emf) replace

        logistic dementia_10 anu_adri if sample_anu_adri==1
        lroc, title("ANU-ADRI") ///
                ytitle("Detection rate") ///
                xtitle("False positive rate") ///
                text(0.85 0.05 "AUC: 0.59 (95% CI 0.58-0.60)", placement(east)) ///
                scheme(s1mono) ///
                xlab(0(0.25)1, nogrid) ylab(0(0.25)1, nogrid)
        graph export "ROC, anu_adri.pdf", as(pdf) replace
        graph export "ROC, anu_adri.emf", as(emf) replace

        logistic dementia_10 age_recruit if sample==1
        lroc, title("Age (years)") ///
                ytitle("Detection rate") ///
                xtitle("False positive rate") ///
                text(0.92 0.02 "AUC: 0.79 (95% CI 0.79-0.80)", placement(east)) ///
                scheme(s1mono) ///
                xlab(0(0.25)1, nogrid) ylab(0(0.25)1, nogrid)
        graph export "ROC, age.pdf", as(pdf) replace
        graph export "ROC, age.emf", as(emf) replace

        ***Graphing scores by dementia status

        *Code adapted from Nick Cox: https://www.statalist.org/forums/forum/general-stata-
discussion/general/1547538-plot-kernel-density-estimates-as-areas
        *visited 5.7.2022

        sort n_eid
```

© 2023 Kivimäki M et al. *JAMA Network Open.*

```
*CAIDE1
tab caide_1
capture drop d0 d1
capture drop xvar
gen xvar = _n - 1 if _n<=16 // based on the theoretical range of 0-15
tab xvar
kdensity caide_1 if dementia_10==0 & sample_caide_1==1, bwidth(1) at(xvar) gen(d0)
kdensity caide_1 if dementia_10==1 & sample_caide_1==1, bwidth(1) at(xvar) gen(d1)
twoway area d1 d0 xvar, color(blue%30 orange%30) ///
        legend(order(2 "No dementia" 1 "Dementia" ) pos(1) ring(0) col(1)) ///
        xtitle("CAIDE Score (Clinical version)") ///
        ytitle("Density") ///
        scheme(s1color)
graph export "Kernel_density, caide_1_upd.pdf", as(pdf) replace
graph export "Kernel_density, caide_1_upd.emf", as(emf) replace

sum caide_1 if sample_caide_1
*observed range 0-14
twoway area d1 d0 xvar if xvar>=0 & xvar<=14, color(blue%30 orange%30) ///
        legend(order(2 "No dementia" 1 "Dementia" ) pos(1) ring(0) col(1)) ///
        xtitle("CAIDE Score (Clinical version)") ///
        ytitle("Density") ///
        scheme(s1color)
graph export "Kernel_density, caide_1_upd_obs.pdf", as(pdf) replace
graph export "Kernel_density, caide_1_upd_obs.emf", as(emf) replace

*CAIDE2
tab caide_2
capture drop d0 d1
capture drop xvar
gen xvar = _n - 1 if _n<=19 // based on the theoretical range of 0-18
tab xvar
kdensity caide_2 if dementia_10==0 & sample_caide_2==1, bwidth(1) at(xvar) gen(d0)
kdensity caide_2 if dementia_10==1 & sample_caide_2==1, bwidth(1) at(xvar) gen(d1)
twoway area d1 d0 xvar, color(blue%30 orange%30) ///
        legend(order(2 "No dementia" 1 "Dementia" ) pos(1) ring(0) col(1)) ///
        xtitle("CAIDE Score (Clinical version)") ///
        ytitle("Density") ///
        scheme(s1color)
graph export "Kernel_density, caide_2_upd.pdf", as(pdf) replace
graph export "Kernel_density, caide_2_upd.emf", as(emf) replace

sum caide_2 if sample_caide_2
*observed range 0-17
twoway area d1 d0 xvar if xvar>=0 & xvar<=17, color(blue%30 orange%30) ///
        legend(order(2 "No dementia" 1 "Dementia" ) pos(1) ring(0) col(1)) ///
        xtitle("CAIDE Score (APOE supplemented)") ///
        ytitle("Density") ///
        scheme(s1color)
graph export "Kernel_density, caide_2_upd_obs.pdf", as(pdf) replace
graph export "Kernel_density, caide_2_upd_obs.emf", as(emf) replace

*BRIEF
tab brief
capture drop d0 d1
capture drop xvar
gen xvar = _n - 1 if _n<=57 // based on the theoretical range of 0-56
tab xvar
kdensity brief if dementia_10==0 & sample_brief==1, bwidth(1) at(xvar) gen(d0)
kdensity brief if dementia_10==1 & sample_brief==1, bwidth(1) at(xvar) gen(d1)
```

© 2023 Kivimäki M et al. *JAMA Network Open.*

```
twoway area d1 d0 xvar, color(blue%30 orange%30) ///
        legend(order(2 "No dementia" 1 "Dementia" ) pos(1) ring(0) col(1)) ///
        xtitle("BDSI") ///
        ytitle("Density") ///
        scheme(s1color) ///
        xscale(range(0 56)) ///
        xlabel(0 (20) 40)
graph export "Kernel_density, brief_upd.pdf", as(pdf) replace
graph export "Kernel_density, brief_upd.emf", as(emf) replace

sum brief if sample_brief==1
*observed range 0-42
twoway area d1 d0 xvar if xvar>=0 & xvar<=42, color(blue%30 orange%30) ///
        legend(order(2 "No dementia" 1 "Dementia" ) pos(1) ring(0) col(1)) ///
        xtitle("BDSI") ///
        ytitle("Density") ///
        scheme(s1color) ///
        xscale(range(0 42)) ///
        xlabel(0 (20) 40)
graph export "Kernel_density, brief_upd_obs.pdf", as(pdf) replace
graph export "Kernel_density, brief_upd_obs.emf", as(emf) replace


*ANU-ADRI
tab anu_adri
capture drop d0 d1
capture drop xvar
gen xvar = _n - 19 if _n<=133 // based on the theoretical range of -18 to 114
tab xvar
kdensity anu_adri if dementia_10==0 & sample_caide_1==1, bwidth(1) at(xvar) gen(d0)
gsort -n_eid
kdensity anu_adri if dementia_10==1 & sample_caide_1==1, bwidth(1) at(xvar) gen(d1)

twoway area d1 d0 xvar, color(blue%30 orange%30) ///
        legend(order(2 "No dementia" 1 "Dementia" ) pos(1) ring(0) col(1)) ///
        xtitle("ANU-ADRI") ///
        ytitle("Density") ///
        scheme(s1color) ///
        xscale(range(-18 114)) ///
        xlabel(-20 (20) 100)
graph export "Kernel_density, anu_adri_upd.pdf", as(pdf) replace
graph export "Kernel_density, anu_adri_upd.emf", as(emf) replace

sum anu_adri if sample_anu_adri==1
*observed range -18 to 28
twoway area d1 d0 xvar if xvar>=-18 & xvar<=28, color(blue%30 orange%30) ///
        legend(order(2 "No dementia" 1 "Dementia" ) pos(1) ring(0) col(1)) ///
        xtitle("ANU-ADRI") ///
        ytitle("Density") ///
        scheme(s1color) ///
        xscale(range(-18 28)) ///
        xlabel(-20 (10) 30)
graph export "Kernel_density, anu_adri_upd_obs.pdf", as(pdf) replace
graph export "Kernel_density, anu_adri_upd_obs.emf", as(emf) replace


*AGE
tab age_recruit if sample==1
capture drop d0 d1
```

© 2023 Kivimäki M et al. *JAMA Network Open.*

```
capture drop xvar
gen xvar = _n + 37 if _n<=36 // based on the observed range of 38 to 73
tab xvar
kdensity age_recruit if dementia_10==0 & sample==1, bwidth(1) at(xvar) gen(d0)
kdensity age_recruit if dementia_10==1 & sample==1, bwidth(1) at(xvar) gen(d1)
tab xvar

twoway area d1 d0 xvar if xvar>=38 & xvar<=73, color(blue%30 orange%30) ///
	legend(order(2 "No dementia" 1 "Dementia" ) pos(1) ring(0) col(1)) ///
	xtitle("Age (years)") ///
	ytitle("Density") ///
	scheme(s1color)
graph export "Kernel_density, age_recruit_upd.pdf", as(pdf) replace
graph export "Kernel_density, age_recruit_upd.emf", as(emf) replace

**FIGURE 2**

*dementia subtype
tab dementia_code
gen dementia_code_3 = substr(dementia_code,1,3)
tab dementia_code_3
gen dementia_type = 1 if inlist(dementia_code_3,"F00","G30")
replace dementia_type = 2 if inlist(dementia_code_3,"F01")
replace dementia_type = 3 if inlist(dementia_code,"F020","G310")
replace dementia_type = 4 if inlist(dementia_code,"F023")
replace dementia_type = 5 if inlist(dementia_code,"F021","F022","F024","F028","G318")
replace dementia_type = 6 if inlist(dementia_code_3,"F03") | inlist(dementia_code,"F051","G311")
tab dementia_type
tab dementia
label define dem_type 1 "AD" 2 "VaD" 3 "FTD" 4 "PDD" 5 "Other specified" 6 "Unspecified"
label values dementia_type dem_type
tab dementia_type

*** Harrel's c for each dementia subtype

putexcel set "FIGURES_TABLES_output.xlsx", sheet("Table 2") modify
local row 5
foreach score in caide_1 caide_2 brief anu_adri age_recruit {
		forvalues i=0/4 {
			di ""
			di "`score'"
			if `i'==0 {
			    tab dementia_10 if sample_`score'==1
				somersd dementia_10 `score' if sample_`score'==1, transf(c)
				matrix m = r(table)
				quietly: putexcel G`row'=m[1,1], nformat(#.00)
				quietly: putexcel H`row'=m[5,1], nformat(#.00)
				quietly: putexcel I`row'=m[6,1], nformat(#.00)
				local ++row
			}
			else {
				tab dementia_type if dementia_type==`i' & dementia_10==1 &
sample_`score'==1
				capture drop type_10
				gen type_10 = dementia_type==`i' & dementia_10==1
				tab type_10 if sample_`score'==1
				somersd type_10 `score' if sample_`score'==1, transf(c)
				matrix m = r(table)
				quietly: putexcel G`row'=m[1,1], nformat(#.00)
				quietly: putexcel H`row'=m[5,1], nformat(#.00)
```

© 2023 Kivimäki M et al. *JAMA Network Open.*

```
					quietly: putexcel I`row'=m[6,1], nformat(#.00)
					local ++row
				}
			}
			local ++row
		}

*** TABLE 3 ***
capture log close
log using "UKB Table 3 for all and by age group 3.log", replace

*Different cut-offs

*Range
foreach group in all 64 65+ {
di "--------------------"
di "Age group: `group'"
di "--------------------"

	preserve
	if "`group'" == "64" {
		drop if age_recruit>=65
		}
	if "`group'" == "65+" {
		drop if age_recruit<65
		}

foreach score in caide_1 caide_2 brief anu_adri age_recruit {
	tab `score' if sample_`score'==1
}
	restore
}

*Analysis
foreach group in all 64 65+ {
di "--------------------"
di "Age group: `group'"
di "--------------------"

	preserve
	if "`group'" == "64" {
		drop if age_recruit>=65
		}
	if "`group'" == "65+" {
		drop if age_recruit<65
		}

putexcel set "FIGURES_TABLES_output.xlsx", sheet("Table 3_`group'") modify

foreach score in caide_1 caide_2 brief anu_adri age_recruit {
	if "`score'"=="caide_1" {
		local row 6
		local cut_offs "6 7 8 9 10 11 12"
		local min 0
	}
	if "`score'"=="caide_2" {
		local row 14
		local cut_offs "6 7 8 9 10 11 12 13 14"
		local min 0
	}
```

© 2023 Kivimäki M et al. *JAMA Network Open.*

```
	if "`score'"=="brief" {
		local row 24
		local cut_offs "5 7 9 10 15 16 22"
		local min 0
	}
	if "`score'"=="anu_adri" {
		local row 32
		local cut_offs "-10 -5 -3 -2 1 5 6 7 10"
		local min -18
	}
	if "`score'"=="age_recruit" {
		local row 42
		local cut_offs "50 55 60 64 65 66 67 68 69 70"
		local min 37
	}

di ""
di "SCORE: `score'"
di ""
tab `score' if sample_`score'==1
sum age_recruit if sample_`score'==1
somersd dementia_10 `score' if sample_`score'==1, transf(c)
matrix c = r(table)
matrix list c

quietly: putexcel Q`row'=c[1,1], nformat(#.00)
quietly: putexcel R`row'=c[5,1], nformat(#.00)
quietly: putexcel S`row'=c[6,1], nformat(#.00)
local ++row

foreach i in `cut_offs' {
	di ""
	di "Cut `score' at <`i' vs `i'+"
	capture drop `score'_cut
	egen `score'_cut = cut(`score'), at(`min',`i',100)
	tab `score'_cut if sample_`score'==1
	tab dementia_10 `score'_cut if sample_`score'==1, matcell(m)
	matrix list m
		quietly: putexcel T`row'=m[2,2], nformat(number)
		quietly: putexcel U`row'=m[2,1], nformat(number)
		quietly: putexcel V`row'=m[1,2], nformat(number)
		quietly: putexcel W`row'=m[1,1], nformat(number)
	tab dementia_10 `score'_cut if sample_`score'==1, row
	local ++row
	}
}
restore
}
capture log close

*** TABLE 3 sensitivity analysis including also those with short follow-up***
capture log close
log using "UKB Table 3 sensitivity analysis including also short follow up broad 3.log", replace

foreach group in sens {
di "--------------------"
di "Age group: `group'"
di "--------------------"

	preserve
```

```
	if "`group'" == "sens" {
		*sensitivity analysis that does not exlcude those with short follow up
		capture drop dementia_10
		stset date_exit if sample_sens==1, failure(dementia) id(n_eid) origin(date_recruit) scale(365.25) 
exit(date_exit_10)
		gen dementia_10 = _d==1
		tab dementia_10
		}

putexcel set "FIGURES_TABLES_output.xlsx", sheet("Table 3_`group'") modify
foreach score in caide_1 caide_2 brief anu_adri age_recruit {
	if "`score'"=="caide_1" {
		local row 6
		local cut_offs "6 7 8 9 10 11 12"
		local min 0
	}
	if "`score'"=="caide_2" {
		local row 14
		local cut_offs "6 7 8 9 10 11 12 13 14"
		local min 0
	}
	if "`score'"=="brief" {
		local row 24
		local cut_offs "5 7 9 10 15 16 22"
		local min 0
	}
	if "`score'"=="anu_adri" {
		local row 32
		local cut_offs "-10 -5 -3 -2 1 5 6 7 10"
		local min -18
	}
	if "`score'"=="age_recruit" {
		local row 42
		local cut_offs "50 55 60 64 65 66 67 68 69 70"
		local min 37
	}

di ""
di "SCORE: `score'"
di ""
tab `score' if sample_sens_`score'==1
sum age_recruit if sample_sens_`score'==1
somersd dementia_10 `score' if sample_sens_`score'==1, transf(c)
matrix c = r(table)
matrix list c

quietly: putexcel Q`row'=c[1,1], nformat(#.00)
quietly: putexcel R`row'=c[5,1], nformat(#.00)
quietly: putexcel S`row'=c[6,1], nformat(#.00)
local ++row

foreach i in `cut_offs' {
	di ""
	di "Cut `score' at <`i' vs `i'+"
	capture drop `score'_cut
	egen `score'_cut = cut(`score'), at(`min',`i',100)
	tab `score'_cut if sample_sens_`score'==1
	tab dementia_10 `score'_cut if sample_sens_`score'==1, matcell(m)
	matrix list m
		quietly: putexcel T`row'=m[2,2], nformat(number)
```

```
			quietly: putexcel U`row'=m[2,1], nformat(number)
			quietly: putexcel V`row'=m[1,2], nformat(number)
			quietly: putexcel W`row'=m[1,1], nformat(number)
		tab dementia_10 `score'_cut if sample_sens_`score'==1, row
		local ++row
		}
}
restore
}
capture log close



*** TABLE 4 at age 65 +/- 1 ***
capture log close
log using "UKB Table 4 (age 65 +- 1) 3.log", replace

*Different cut-offs

foreach group in all {
di "--------------------"
di "Age group: `group'"
di "--------------------"

		preserve
		keep if inlist(age_recruit,64,65,66)

putexcel set "FIGURES_TABLES_output.xlsx", sheet("Table 4") modify
foreach score in caide_1 caide_2 brief anu_adri age_recruit {
		if "`score'"=="caide_1" {
			local row 6
			local cut_offs "6 7 8 9 10 11 12"
			local min 0
		}
		if "`score'"=="caide_2" {
			local row 14
			local cut_offs "6 7 8 9 10 11 12 13 14"
			local min 0
		}
		if "`score'"=="brief" {
			local row 24
			local cut_offs "5 7 9 10 15 16 22"
			local min 0
		}
		if "`score'"=="anu_adri" {
			local row 32
			local cut_offs "-10 -5 -3 -2 1 5 6 7 10"
			local min -18
		}

di ""
di "SCORE: `score'"
di ""
tab `score' if sample_`score'==1
sum age_recruit if sample_`score'==1
somersd dementia_10 `score' if sample_`score'==1, transf(c)
matrix c = r(table)
matrix list c

quietly: putexcel Q`row'=c[1,1], nformat(#.00)
```

```
quietly: putexcel R`row'=c[5,1], nformat(#.00)
quietly: putexcel S`row'=c[6,1], nformat(#.00)
local ++row

foreach i in `cut_offs' {
	di ""
	di "Cut `score' at <`i' vs `i'+"
	capture drop `score'_cut
	egen `score'_cut = cut(`score'), at(`min',`i',100)
	tab `score'_cut if sample_`score'==1
	tab dementia_10 `score'_cut if sample_`score'==1, matcell(m)
	matrix list m
		quietly: putexcel T`row'=m[2,2], nformat(number)
		quietly: putexcel U`row'=m[2,1], nformat(number)
		quietly: putexcel V`row'=m[1,2], nformat(number)
		quietly: putexcel W`row'=m[1,1], nformat(number)
	tab dementia_10 `score'_cut if sample_`score'==1, row
	local ++row
	}
}
restore
}
capture log close


*** ANALYSES FOR THE MANUSCRIPT ***
capture log close
log using "UKB additional analyses.log", replace

tab dementia_10 if sample==1
stdescribe if sample==1
strate if sample==1, per(10000)

*Create quintiles:
foreach score in caide_1 caide_2 brief anu_adri age_recruit {
	capture drop `score'_quint
	tab `score' if sample_`score'==1
	centile `score' if sample_`score'==1, centile(20(20)80)
	xtile `score'_quint = `score' if sample_`score'==1, nq(5)
	tab `score'_quint
	tab `score'_quint dementia_10
	bysort `score'_quint: sum `score'
	}

*Cox models for quintiles
foreach score in caide_1 caide_2 brief anu_adri age_recruit {
	di ""
	di "Cox model: Quintiles of `score' and the 10-year risk of dementia"
	tab `score'_quint if sample_`score'==1
	tab `score'_quint _d if sample_`score'==1, row
	stcox i.`score'_quint if sample_`score'==1, strata(area_0_0) base
	}

log close


**** WHITEHALL II STUDY ****

capture log close
log using "Analysis WHII 02012023 caide phase 5.log", replace
```

© 2023 Kivimäki M et al. *JAMA Network Open.*

```
use "WHII_dementia_risk_scores_caide_phase5.dta", clear

*Put dementia to missing if dementia diagnosis was recorded after the end of the follow-up
count if dementia_date > date_exit & dementia_date<.
*0 to missing

*Flowchart

*Attended baseline assessment
*10 308 (e.g. Machado-Fragua 2022 Circulating serum metabolites as predictors of dementia OR Lindbohm 2019 5-
year.... OR https://www.ucl.ac.uk/epidemiology-health-care/research/epidemiology-and-public-
health/research/whitehall-ii/data-collection, visited 17112022)

*Attendance, phase 5: 7,870 (https://www.ucl.ac.uk/epidemiology-health-care/research/epidemiology-and-public-
health/research/whitehall-ii/data-collection, visited 17112022)
sum tdatscrn
*7870, this is correct
capture drop phase5
gen phase5 = tdatscrn!=.
tab phase5

*Attending clinical screening at phase5
capture drop phase5_clinical
gen phase5_clinical = age_s5!=.
tab phase5_clinical
*6551, this is close to 6537, which was reported by Machado-Fragua et al. 2022 Circulating serum metabolites as
predictors of dementia

di 10308-6551
*3757 did not attend phase 5 clinical examination

*Reasons for non-attendance
*died before phase 5
gen death_s5 = date_death<=tdatscrn & date_death!=.
tab death_s5
tab death_s5 phase5_clinical

*Non-attendance for other reason
tab phase5_clinical death_s5, row
di 2958/10308
*28.7%

*Missing data for each risk score:

*CAIDE1:
capture drop full_data_caide_1
gen full_data_caide_1 = 1
foreach var in age_caide_1 education_caide_1 sex_caide_1 sbp_caide_1 bmi_caide_1 tot_chol_caide_1
phyact_caide_1 tdatscrn date_exit {
        replace full_data_caide_1 = 0 if `var'==.
}
tab full_data_caide_1
*4872 yes
tab phase5_clinical full_data_caide_1
tab phase5_clinical phase5 if full_data_caide_1==0
*5436 no (2438 did not attend phase 5 at all, 1319 did not attend phase 5 clinical assessment, 1679 had missing data)
di 6551-1679
di 1679/6551
*25.6%
```

© 2023 Kivimäki M et al. *JAMA Network Open.*

```
*CAIDE2:
gen full_data_caide_2 = 1
foreach var in age_caide_1 education_caide_1 sex_caide_1 sbp_caide_1 bmi_caide_1 tot_chol_caide_1
phyact_caide_1 apoe_caide_2 tdatscrn date_exit {
	replace full_data_caide_2 = 0 if `var'==.
}
tab full_data_caide_2
*3785 yes
tab phase5_clinical full_data_caide_2
tab phase5_clinical phase5 if full_data_caide_2==0
*6523 no (2438 did not attend phase 5 at all, 1319 did not attend phase 5 clinical assessment, 2766 had missing data)

*Dementia at baseline
capture drop dementia_baseline
gen dementia_baseline = dementia_date<=tdatscrn & tdatscrn<.
tab dementia_baseline
*0

*Follow up less than 365 days
gen short_fu = (date_exit - tdatscrn)<365
tab short_fu if full_data_caide_1
*7

*Theoretical follow-up less than 10 years (theoretical follow up is until 21824 [02oct2019])
capture drop theor_fu
gen theor_fu = (21824 - tdatscrn)/365.25
capture drop theor_fu_10
sum theor_fu
egen theor_fu_10 = cut(theor_fu), at(0,10,100)
tab theor_fu_10
*0

*Theoretical follow-up less than 20 years
capture drop theor_fu_20
sum theor_fu
egen theor_fu_20 = cut(theor_fu), at(0,20,100)
tab theor_fu_20
*0

*GENERATE VARIABLES FOR ANALYTIC SAMPLES
foreach score in caide_1 caide_2 {
capture drop sample_`score'
gen sample_`score' = full_data_`score'==1
replace sample_`score' = 0 if dementia_baseline==1
replace sample_`score' = 0 if short_fu==1
replace sample_`score' = 0 if theor_fu<10
tab sample_`score'
}
gen sample = sample_caide_1
gen sample_age_s5_full = sample_caide_1

*GENERATE DEMENTIA RISK SCORES
gen caide_1 = age_caide_1 + education_caide_1 + sex_caide_1 + sbp_caide_1 + bmi_caide_1 + tot_chol_caide_1 +
phyact_caide_1
tab sample_caide_1
sum caide_1 if sample_caide_1==1

gen caide_2 = age_caide_2 + education_caide_2 + sex_caide_2 + sbp_caide_2 + bmi_caide_2 + tot_chol_caide_2 +
phyact_caide_2 + apoe_caide_2
```

© 2023 Kivimäki M et al. *JAMA Network Open.*

```
tab sample_caide_2
sum caide_2 if sample_caide_2==1

tab sample_caide_1 sample_caide_2, mis
tab sample

**** 10-year risk ****
capture drop date_exit_10
capture drop dementia_10
gen date_exit_10 = tdatscrn + round(10*365.25)
stset date_exit if sample==1, failure(dementia) id(ID_OUT) origin(tdatscrn) scale(365.25) exit(date_exit_10)
gen dementia_10 = _d==1
tab dementia_10
tab sample_caide_1 dementia_10
*17 dementia cases
tab sample_caide_2 dementia_10
*10 dementia cases

**** 20-year risk ****
capture drop date_exit_20
capture drop dementia_20
gen date_exit_20 = tdatscrn + round(20*365.25)
stset date_exit if sample==1, failure(dementia) id(ID_OUT) origin(tdatscrn) scale(365.25) exit(date_exit_20)
gen dementia_20 = _d==1
tab dementia_20

tab sample_caide_1 dementia_20
*202 dementia cases
tab sample_caide_2 dementia_20
*145 dementia cases


***** TABLE 1 ***************

			*20-year dementia risk
			sum caide_1 caide_2 if sample==1
			bysort dementia_20: sum caide_1 caide_2 if sample==1

			baselinetable ///
			age_s5_full(cts novarlabel afterhead("Age at entry, years")) ///
			sex(novarlabel afterhead("Sex")) ///
			non_white(novarlabel afterhead("Non-white ethnicity")) ///
			caide_1(cts novarlabel afterhead("CAIDE score (clinical version)")) ///
			caide_2(cts novarlabel afterhead("CAIDE score (APOE supplemented)")) ///
			if sample==1 ///
			, by(dementia_20, total) ctsvartab(mean (sd)) missing ///
			exportexcel(Table_1_WHII_p5_20y, cell(A6) replace)

*P-values for Table 1
		ttest age_s5_full if sample==1, by(dementia_20)
		tab sex dementia_20 if sample==1, chi
		tab non_white dementia_20 if sample==1, chi
		ttest caide_1 if sample==1, by(dementia_20)
		ttest caide_2 if sample==1, by(dementia_20)

*** TABLE 5 (equivalent of Table 3 of the UK Biobank analysis) ***

*Different cut-offs

putexcel set "FIGURES_TABLES_output.xlsx", sheet("Table 3 WHII p5") modify
```

© 2023 Kivimäki M et al. *JAMA Network Open.*

```
foreach fu in 10 20 {
foreach score in caide_1 caide_2 age_s5_full {
	if "`score'"=="caide_1" {
		local row 8
		local cut_offs "6 7 8 9 10 11 12"
		local min 0
	}
	if "`score'"=="caide_2" {
		local row 16
		local cut_offs "6 7 8 9 10 11 12 13 14"
		local min 0
	}
	if "`score'"=="age_s5_full" {
		local row 26
		local cut_offs "50 55 60 64 65 66 67 68"
		local min 45
	}

if "`fu'"=="20" {
   local row = `row' + 28
}

di ""
di "SCORE: `score'"
di ""
tab `score' if sample_`score'==1
sum age_s5_full if sample_`score'==1
somersd dementia_`fu' `score' if sample_`score'==1, transf(c)
matrix c = r(table)
matrix list c

quietly: putexcel Q`row'=c[1,1], nformat(#.00)
quietly: putexcel R`row'=c[5,1], nformat(#.00)
quietly: putexcel S`row'=c[6,1], nformat(#.00)
local ++row

foreach i in `cut_offs' {
	di ""
	di "Cut `score' at <`i' vs `i'+"
	capture drop `score'_cut
	egen `score'_cut = cut(`score'), at(`min',`i',100)
	tab `score'_cut if sample_`score'==1
	tab dementia_`fu' `score'_cut if sample_`score'==1, matcell(m)
	matrix list m
		quietly: putexcel T`row'=m[2,2], nformat(number)
		quietly: putexcel U`row'=m[2,1], nformat(number)
		quietly: putexcel V`row'=m[1,2], nformat(number)
		quietly: putexcel W`row'=m[1,1], nformat(number)
	tab dementia_`fu' `score'_cut if sample_`score'==1, row
	local ++row
	}
}
}

log close
```

© 2023 Kivimäki M et al. *JAMA Network Open.*
