# Supplementary material for: Estimating Dementia Risk Using Multifactorial Prediction Models
Source: JAMA Netw Open. 2023 Jun 13;6(6):e2318132. doi: 10.1001/jamanetworkopen.2023.18132 (PMC10265307; doi:10.1001/jamanetworkopen.2023.18132)
Supplement: Supplement 2. — Data Sharing Statement [file jamanetwopen-e2318132-s002.pdf]

Data Sharing Statement

Kivimäki. Estimating Dementia Risk Using Multifactorial Prediction Models. *JAMA Netw Open*. Published June 13, 2023. doi:10.1001/jamanetworkopen.2023.18132

Data
Data available: Yes
Data types: Deidentified participant data
How to access data: These data are available from UK Biobank: https://www.ukbiobank.ac.uk/enable-your-research/apply-for-access
When available: beginning date: 03-01-2023

Supporting Documents
Document types: Statistical/analytic code
How to access documents: Statistical code is provided in manuscript supplementary appendix
When available: With publication

Additional Information
Who can access the data: For bona fide researchers
Types of analyses: For research purposes
Mechanisms of data availability: With a signed data access agreement
